# Emerging Therapeutic Perspectives in Obese Patients with MASLD Leading to Compensated Advanced Chronic Liver Disease

**DOI:** 10.3390/biom16060797

**Published:** 2026-05-28

**Authors:** Roberta Chianetta, Lydia Giannitrapani, Alessio Giuseppe Lipari, Assunta Brunone, Claudia Cannizzo, Roberto Citarrella, Maurizio Soresi, Antonio Liguori, Nadia Panera, Filomena Morisco, Luca Miele, Anna Licata

**Affiliations:** 1 Medicina Interna, AOUP “P. Giaccone”, PROMISE, Università di Palermo, 90127 Palermo, Italy; chianetta.roberta8@gmail.com (R.C.); lydia.giannitrapani@unipa.it (L.G.); alessiolipari99@gmail.com (A.G.L.); assunta.brunone@gmail.com (A.B.); claudiacannizzo24@gmail.com (C.C.); roberto.citarrella@unipa.it (R.C.); maurizio.soresi@unipa.it (M.S.); 2 IRCCS Ospedale Pediatrico Bambino Gesù, Università Cattolica del Sacro Cuore, 00165 Rome, Italy; antonio.liguori@policlinicogemelli.it (A.L.); luca.miele@policlinicogemelli.it (L.M.); 3 Research Unit of Genetics of Complex Phenotypes, “Bambino Gesù” Children’s Hospital, IRCCS, 00165 Rome, Italy; nadia.panera@opbg.net; 4 UO di Gastroenterologia, Università Federico II, 80131 Naples, Italy; filomena.morisco@unina.it

**Keywords:** MASLD, obesity, cACLD, SGLT-2I, GLP-1 RA, GLP1/G

## Abstract

Metabolic dysfunction-associated steatotic liver disease (MASLD) is now recognized as the principal hepatic manifestation of obesity and metabolic dysfunction. Its pathogenesis is complex and multifactorial, driven by insulin resistance, low-grade chronic inflammation, oxidative stress, gut microbiota alterations, and abnormalities in lipid metabolism; together, these promote steatosis, lipotoxicity, and progression to fibrosis which can lead to compensated advanced chronic liver disease (cACLD). MASLD is also a multisystem condition closely associated with an increased risk of major adverse cardiovascular events such as myocardial infarction, ischemic stroke, atrial fibrillation, and other extrahepatic complications. In this context, emerging metabolic therapies show significant potential for modifying the natural history of the disease. Glucagon-like peptide (GLP)-1 receptor agonists induce substantial weight loss and improve steatosis and necro-inflammatory activity. Sodium–glucose cotransporter 2 inhibitors (SGLT-2I) reduce glucotoxicity, promote modest weight loss, and lower hepatic fat content by improving insulin sensitivity and inflammatory signaling. Even more promising are dual GLP-1/GIP receptor agonists, which have demonstrated superior efficacy in metabolic control, reducing hepatic steatosis, and potentially modulating fibrotic processes, although definitive histological confirmation is still lacking. Overall, in this review, we discuss the physiopathological mechanisms of MASLD leading to cACLD along with the emerging therapies, such GLP1 receptor agonists, SGLT-2I, and GLP1/GIP which, when combined with structured lifestyle interventions, may attenuate progression toward steatohepatitis (MASH), fibrosis, and, thus, cirrhosis.

## 1. The Burden of MASLD

Metabolic dysfunction-associated steatotic liver disease (MASLD) is the most prevalent cause of chronic liver disease worldwide and is the principal hepatic manifestation of obesity and metabolic dysfunction. It is defined by the presence of intrahepatic lipid accumulation in association with cardiometabolic abnormalities, in the absence of other causes of liver steatosis. The pathophysiological mechanisms involved are complex and include insulin resistance (IR), dysregulation of lipid and glucose metabolism, chronic low-grade inflammation, oxidative stress, and alterations in the gut–liver axis. Taken together, these mechanisms are involved in the progression of liver disease from simple steatosis to more advanced stages of fibrosis, ultimately leading to compensated advanced chronic liver disease (cACLD) [1].

Emerging evidence indicates that MASLD is not merely a liver disease but a systemic cardiometabolic disorder in which hepatic dysfunction, IR, and adipose tissue dysregulation collectively contribute to increased cardiovascular risk, chronic kidney disease, and other metabolic comorbidities, substantially impacting patient morbidity and mortality. The conceptual shift from NAFLD to MASLD highlights the central role of metabolic dysfunction in disease pathogenesis and supports a more integrated clinical approach [2]. Although a large body of evidence supports a strong association between MASLD and cardiovascular as well as systemic complications, most available data derive from observational studies and are therefore subject to residual confounding by shared cardiometabolic risk factors, including obesity, IR, and hypertension.

Consequently, while MASLD is increasingly recognized as a potential contributor to cardiometabolic risk, a direct causal relationship remains difficult to establish, and the observed associations likely reflect a complex interplay between hepatic and systemic metabolic dysfunction.

Therapeutic strategies targeting underlying metabolic mechanisms, combined with structured lifestyle interventions, are a promising path toward preventing progression to steatohepatitis and advanced fibrosis and reducing both hepatic and extrahepatic complications.

MASLD currently affects approximately 30% of the general adult population, with its prevalence increasing substantially in individuals with metabolic disorders. Rates exceed 55% in patients with type 2 diabetes mellitus (T2DM) and reach around 65% in those with concomitant obesity and T2DM [3,4]. It is strongly associated with obesity, IR, dyslipidemia, hypertension, and metabolic syndrome (MetS), all of which influence disease onset, progression, and clinical outcomes [3,4,5].

Sex- and gender-specific differences in prevalence, clinical manifestations, and therapeutic responses are increasingly recognized in chronic liver disease, especially of metabolic origin. These differences likely result from a complex interplay of biological and hormonal factors, pharmacokinetic variations, and sociocultural determinants. Lower adherence to and persistence of long-term pharmacological treatments have been reported more frequently among women, with potential implications for disease progression and outcomes [6,7,8]. A better characterization of sex-specific differences could improve risk stratification and support personalized diagnostic and therapeutic approaches, particularly in patients with MASLD and concomitant T2DM [9]. In this perspective, we decided to review the current literature to analyze the treatments available to patients with MASLD, obesity and T2DM who could be exposed to cardio and cerebrovascular events, but above all to the progression of chronic liver disease.

## 2. Literature Search Strategy

This narrative review was based on a comprehensive literature search aimed at identifying relevant studies on MASLD, with a particular focus on pathophysiology, cardiovascular risk, and emerging pharmacological therapies.

A structured search of the literature was conducted using major electronic databases, including PubMed/MEDLINE, Scopus, and Web of Science. The search strategy combined relevant keywords such as “MASLD”, “NAFLD”, “insulin resistance”, “cardiovascular risk”, “SGLT2 inhibitors”, “GLP-1 receptor agonists”, and “dual incretin agonists”.

Preference was given to high-quality evidence, including guidelines, randomized controlled trials, meta-analyses, and large observational studies published in English. Additional relevant publications were identified through a manual search of the reference lists of the selected articles.

Given the narrative nature of this review, studies were selected based on their relevance to the topic and their contribution to the understanding of the pathophysiological mechanisms involved and clinical implications.

## 3. From Insulin Resistance and Type 2 Diabetes to MASLD

MASLD is the hepatic manifestation of systemic cardiometabolic dysfunction, with IR as the primary determinant. At the molecular level, IR arises from dysregulated IRS/PI3K–AKT signaling leading to compensatory hyperinsulinemia, increased hepatic glucose production, impaired suppression of adipose tissue lipolysis, and ectopic lipid accumulation in liver and muscle tissue [10,11,12]. These alterations create a pro-atherogenic metabolic milieu linking hepatic IR directly to cardiovascular risk.

Clinical indices such as the Homeostasis Model Assessment of Insulin Resistance (HOMA-IR) correlate intrahepatic triglyceride content with the histologic severity of MASLD, while adipose tissue insulin resistance in T2DM is associated with fibrosis progression [13,14]. In hepatocytes, chronic hyperinsulinemia induces “selective insulin resistance”: metabolic signaling is impaired, while lipogenic pathways remain active. This activates Sterol Regulatory Element-Binding Protein 1c (SREBP-1c) and Carbohydrate-Responsive Element-Binding Protein (ChREBP), inducing de novo lipogenesis (DNL) via Acetyl-CoA Carboxylase (ACC), Fatty Acid Synthase (FASN), and Stearoyl-CoA Desaturase 1 (SCD1) [15,16]. Stable isotope tracer studies show that DNL contributes up to 25–30% of intrahepatic triglycerides in MASLD patients, whereas dietary interventions and weight loss reduce DNL and liver fat as well as improve insulin sensitivity [17,18]. Increased DNL, combined with insufficient Very Low-Density Lipoprotein (VLDL) secretion, promotes an atherogenic lipid profile: hypertriglyceridemia, small dense Low-Density Lipoprotein (LDL), and reduced High-Density Lipoprotein (HDL) [19,20]. The loss of insulin’s anti-lipolytic effect in visceral adipose tissue increases the flux of free fatty acids (FFAs) to the liver, accounting for roughly two-thirds of intrahepatic triglycerides. The accumulation of bioactive lipid intermediates, including DAG (diacylglycerols) and ceramides, further inhibits insulin signaling (PKCε/AKT), aggravating hepatic IR [21,22]. Mitochondrial overload induces incomplete β-oxidation, excessive reactive oxygen/nitrogen species (ROS/RNS) production, and lipid peroxidation, forming malondialdehyde (MDA), thiobarbituric acid-reactive substances (TBARSs), and F2α-isoprostanes, which correlate with disease severity and fibrosis [23,24,25,26]. Lipotoxicity and exposure to intestinal endotoxins activate innate immune pathways. FFAs and lipotoxic intermediates promote inflammatory signaling through TNF-α-mediated IKKβ/NF-κB activation. In parallel, the activation of the NLRP3 inflammasome induces caspase-1-dependent maturation of IL-1β and IL-18, contributing to IR, hepatic inflammation, and fibrosis progression [27,28]. Intestinal dysbiosis and increased lipopolysaccharide (LPS) activate TLR4 in hepatic stellate cells (HSC), suppressing BMP and Activin Membrane-Bound Inhibitor (BAMBI) and amplifying Transforming Growth Factor β (TGF-β)-mediated fibrogenesis [29,30,31]. Microbial metabolites, including endogenous ethanol and acetate, increase hepatic acetyl-CoA, stimulate DNL, and worsen oxidative stress [32,33,34,35].

**Figure 1:**
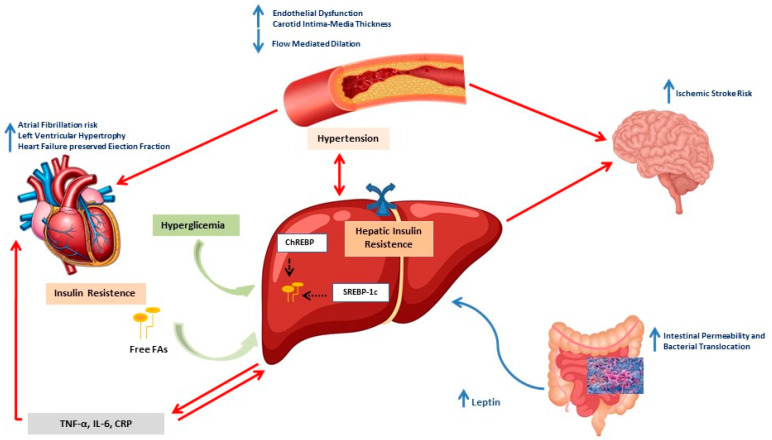
Pathophysiological Mechanisms in MASLD and Cardio and Cerebrovascular Risk. Clinical–pathophysiological interplay between insulin resistance, hepatic metabolic dysfunction, and hypertension, mediated by ChREBP (Carbohydrate Response Element-Binding Protein) and SREBP-1c (Sterol Regulatory Element-Binding Protein 1c), promotes steatosis, endothelial dysfunction, cardiac remodeling, and increased risk of atrial fibrillation and ischemic stroke. The gut microbiota, through intestinal permeability and leptin, contributes to inflammation and metabolic dysregulation.

These alterations are not confined to the liver: clinical and imaging studies demonstrate that MASLD is associated with endothelial dysfunction, arterial stiffness, and subclinical atherosclerosis, independent of traditional risk factors [36,37,38]. An insulin-resistant liver acts as both the target and amplifier of systemic metabolic dysfunction, orchestrating atherogenic lipoprotein remodeling, inflammation, oxidative stress, and pro-thrombotic imbalance (Figure 1). At this point, MASLD emerges as a fundamental determinant of cardiovascular risk, requiring an integrated approach to cardiometabolic risk stratification and management.

## 4. Visceral Adiposity and Obesity in MASLD

Alongside mechanisms related to IR and T2DM, obesity—particularly excess visceral adipose tissue—is an upstream determinant of MASLD, acting through tightly interconnected metabolic, inflammatory, and fibrogenic pathways. Visceral adiposity increases the release of FFAs into the portal circulation, exacerbating hepatic lipid accumulation and activating pro-inflammatory pathways which not only drive MASLD progression, but also contribute to endothelial dysfunction, arterial stiffness, and systemic atherogenesis, thus connecting obesity and cardiovascular risk [38]. Adipose tissue in obesity is characterized by macrophage infiltration and a chronic pro-inflammatory state which alters adipokine secretion and contributes to systemic metabolic dysfunction [31].

Reduced adiponectin impairs AdipoR1–AMPK and AdipoR2–PPARα signaling, reducing fatty acid oxidation and contributing to hepatic and systemic IR. Excess leptin promotes inflammation and fibrosis through HSC activation and osteopontin expression. Hyperinsulinemia and hyperglycemia resulting from obesity-induced IR amplify hepatic fibrosis via CTGF upregulation, while adipokine imbalance further activates pro-atherogenic pathways, including IRS serine phosphorylation, suppressor of cytokine signaling 3 (SOCS3) induction, and NLRP3 inflammasome activation, linking liver pathology directly to cardiovascular risk [39,40].

This vicious cycle between obesity, metabolic dysfunction, and liver injury accelerates MASLD progression to steatohepatitis and advanced fibrosis. The same mechanisms increase cardiovascular risk: reduced adiponectin and excess leptin promote endothelial dysfunction, vascular inflammation, and myocardial fibrosis, while chronic hyperinsulinemia, hyperglycemia, and lipotoxicity accelerate atherogenesis and arterial stiffness. Systemic inflammation from obesity and MASLD amplifies atherosclerotic plaque formation and destabilization, increasing the risk of myocardial infarction, stroke, and heart failure [10,36,39].

Furthermore, sarcopenia and sarcopenic obesity contribute to MASLD pathophysiology and cardiovascular risk by promoting IR, impaired energy metabolism, and pro-inflammatory signaling [41,42,43]. Pediatric studies report MASLD prevalence in up to 70% of obese adolescents, indicating that early adiposity is sufficient to trigger hepatic metabolic alterations [44]. Studies using hyperinsulinemic euglycemic clamps and magnetic resonance imaging (MRI)-based fat quantification confirm that hepatic IR is tightly associated with intrahepatic triglyceride content and early mitochondrial dysfunction, providing mechanistic links between metabolic impairment and cellular injury in MASLD and systemic cardiometabolic risk [45].

The combined effects of IR, visceral adiposity, and adipokine dysregulation create a systemic pro-inflammatory and pro-atherogenic environment, setting the stage for cardiovascular complications [46]. All together, these mechanisms underline that MASLD is a systemic cardiometabolic condition in which liver disease reflects and amplifies systemic metabolic dysfunction, requiring integrated hepatic and cardiovascular assessment and management.

## 5. Pathophysiological Mechanisms Leading from MASLD to cACLD

In patients with MASLD who progress to cACLD, the metabolic and inflammatory mechanisms described above are thought to converge with the structural and hemodynamic alterations that characterize advanced fibrosis. While cACLD remains clinically compensated, it is defined by significant architectural distortion and increased intrahepatic vascular resistance, which represents the substrate for clinically significant portal hypertension (CSPH) [47,48]. In obese individuals, persistent metabolic injury amplifies these processes through sinusoidal endothelial dysfunction, extracellular matrix (ECM) remodeling, and heightened intrahepatic vasoconstriction [49].

Chronic exposure to hyperinsulinemia, lipotoxic mediators, and systemic inflammation is believed to promote the sustained activation of HSCs, leading to progressive collagen deposition and sinusoidal capillarization [50]. In parallel, reduced nitric oxide (NO) bioavailability—secondarily to oxidative stress and endothelial dysfunction—impairs sinusoidal vasodilation while increased endothelin-1 levels and the activation of the renin–angiotensin–aldosterone system (RAAS) increase intrahepatic vasoconstriction [51,52]. These dynamic components contribute significantly to elevated portal pressure beyond the fixed structural component of fibrosis [48]. In obesity, this imbalance is further exacerbated by systemic endothelial dysfunction and arterial stiffness, reinforcing the bidirectional liver–cardiovascular axis [53,54].

Adipose tissue-derived mediators continue to play a central role in cACLD. Leptin may sustain fibrogenic signaling and angiogenesis, whereas hypoadiponectinemia may reduce antifibrotic and vasoprotective effects [55]. Moreover, the prothrombotic milieu associated with obesity—characterized by increased plasminogen activator inhibitor-1 (PAI-1), fibrinogen, and platelet activation—has been proposed as a contributor to intrahepatic microthrombosis, a mechanism increasingly recognized in fibrotic progression and portal hypertension [56,57,58,59,60]. This concept aligns with the “parenchymal extinction” hypothesis whereby recurrent microvascular injury may accelerate architectural remodeling [58].

Gut–liver axis alterations persist and may intensify in cACLD. Increased intestinal permeability and bacterial translocation may sustain chronic toll-like receptor signaling, perpetuating low-grade inflammation even in compensated stages [60]. This inflammatory milieu is thought to contribute to ongoing fibrogenesis and may predispose patients to acute decompensation under additional stressors [61]. Importantly, obesity-related sarcopenia and myosteatosis have been associated with worse prognosis, potentially through the impairment of metabolic reserve, altered ammonia handling, and increasing susceptibility to complications [62,63].

Overall, in patients with cACLD, portal hypertension should be considered the result of a complex scenario involving structural fibrosis and dynamic factors, including metabolic dysfunction, endothelial impairment, inflammation, and prothrombotic imbalance [64,65]. Even in the compensated phase, these patients may exhibit an increased risk of progression to decompensation and increased cardiovascular risk, supporting the need for integrated metabolic, hepatic, and cardiovascular risk stratification and management, although dedicated studies in this population remain limited.

## 6. Cardio and Cerebrovascular Risk in MASLD

As previously described, MASLD is a central component within the network of cardiometabolic disease and is strongly associated with increased risks of morbidity and mortality [66]. It should be emphasized that most evidence linking MASLD to cardiovascular and cerebrovascular outcomes derives from observational studies; therefore, while robust associations have been consistently reported, causality remains unproven and residual confounding cannot be excluded. Although MASLD shares several cardiometabolic risk factors with cardiovascular disease (CVD)—including IR, dyslipidemia, obesity, arterial hypertension, and chronic low-grade inflammation—emerging evidence suggests that MASLD is associated with cardiovascular risk, although disentangling its independent contribution from their shared risk factors remains challenging [67,68]. In this context, hepatic steatosis and, more prominently, progressive liver fibrosis promote a pro-atherogenic and prothrombotic milieu characterized by endothelial dysfunction, oxidative stress, platelet activation, and chronic inflammation. This environment is further amplified by dysregulated hepatokine and adipokine signaling, including increased fetuin-A, leptin, and pro-inflammatory cytokines [69,70].

Endothelial dysfunction is one of the earliest cardiovascular abnormalities associated with MASLD. Impaired flow-mediated dilation (FMD), reduced nitric oxide bioavailability, increased carotid intima–media thickness (IMT), and enhanced arterial stiffness are consistently observed, reflecting early vascular remodeling and accelerated vascular aging even in the absence of overt CVD [71]. According to the 2023 ESC/ESH guidelines, increased IMT, carotid plaques, and arterial stiffness are established markers of hypertension-mediated organ damage and are recommended for cardiovascular risk stratification [72,73]. Vascular alterations in MASLD are closely linked to the development and progression of arterial hypertension, which in turn may accelerate hepatic disease progression through the activation of the Renin–Angiotensin–Aldosterone System (RAAS), increased arterial stiffness, and profibrotic signaling, thereby reinforcing the bidirectional liver–vascular axis [74].

Beyond vascular involvement, MASLD is associated with early and progressive cardiac structural and functional abnormalities that are linked to its extrahepatic cardiovascular burden. Echocardiographic studies consistently demonstrate subclinical left ventricular (LV) remodeling, including concentric LV hypertrophy, increased LV mass index, left atrial enlargement, and impaired diastolic relaxation, frequently in the presence of preserved ejection fraction [75,76,77]. While conventional echocardiography may underestimate early myocardial involvement, advanced techniques such as tissue Doppler imaging and speckle-tracking echocardiography allow the detection of subtle myocardial dysfunctions [78,79]. Reduced global longitudinal strain (GLS) has emerged as a sensitive marker of early systolic impairment, suggesting the presence of a MASLD-associated cardiomyopathic phenotype driven by metabolic dysfunction, inflammation, and myocardial steatosis [80]. Several clinical studies have provided mechanistic and prognostic insights into these echocardiographic findings. Using TDI, Goland et al. demonstrated that patients with MASLD but without comorbidities (obesity, hypertension, diabetes) already present early LV diastolic dysfunction, characterized by reduced E’ velocity and altered deceleration time, despite preserved systolic function [81]. Similarly, Fotbolcu et al. reported both diastolic and systolic abnormalities in non-diabetic, normotensive patients with MASLD [82]. Studies comparing hypertensive patients with and without MASLD found that diastolic dysfunction is more prevalent in MASLD, even when systolic parameters are comparable [83]. Using 2D-STE, Karabay et al. identified subclinical LV systolic dysfunction with preserved EF, likely linked to IR [84], while VanWagner et al. showed that MASLD is associated with progressive LV remodeling, impaired longitudinal systolic function, elevated LV filling pressures, and increased left atrial volume, independently of metabolic comorbidities [85,86]. Cardiac magnetic resonance imaging (MRI) studies have further confirmed increased myocardial, epicardial, and pericardial fat accumulation in MASLD, which correlates with structural and functional alterations of the LV [87,88]. Pediatric and adolescent studies have also demonstrated early systolic and diastolic impairment correlated with liver disease severity, highlighting cardiovascular involvement from a young age [89,90,91]. Importantly, LV dysfunction can be detected even in the early stages of MASLD and in individuals without overt CVD or traditional risk factors. Cross-sectional and longitudinal studies [77,85] demonstrate progressive LV remodeling and worsening diastolic function over time, supporting the concept of a dynamic myocardial process rather than a static structural abnormality. These alterations, together with increased myocardial stiffness and systemic inflammation, provide a mechanistic link between MASLD and heart failure with preserved ejection fraction (HFpEF), a clinical entity increasingly recognized in patients with metabolic liver disease.

Left atrial enlargement and dysfunction, reflecting chronically elevated LV filling pressures, further connect myocardial involvement to increased susceptibility to atrial arrhythmias [84,85,86]. Pediatric and adolescent studies consistently report subclinical systolic and diastolic dysfunction in obese individuals with MASLD with myocardial impairment correlating with histological liver severity, suggesting that cardiovascular involvement may begin early in the disease course [89,90,91].

MASLD has emerged as an independent risk factor for atrial fibrillation (AF). Large meta-analyses and population-based studies report a significantly increased long-term incidence of AF even after adjustment for established cardiovascular risk factors [92,93]. Proposed mechanisms include atrial structural remodeling, myocardial fibrosis, autonomic imbalance, and elevated left atrial pressures secondary to LV diastolic dysfunction. Systemic inflammation—reflected by increased circulating levels of TNF-α, IL-6, and C-reactive protein (CRP)—further contributes to atrial fibrogenesis, autonomic dysfunction, and electrical instability, thereby lowering the threshold for AF initiation and maintenance [94,95].

MASLD is also associated with an increased risk of ischemic stroke through both AF-dependent and AF-independent pathways. Observational studies and meta-analyses consistently demonstrate higher cerebrovascular event rates in individuals with MASLD even after adjustment for AF and traditional risk factors, with liver fibrosis emerging as a particularly strong predictor of stroke risk [96,97]. Endothelial dysfunction, accelerated carotid atherosclerosis, increased arterial stiffness, platelet hyperreactivity, and a systemic prothrombotic state likely contribute to cerebrovascular risk beyond the presence of arrhythmias alone [96]. At the mechanistic level, myocardial dysfunction in MASLD appears to be driven by a complex interplay of IR, chronic inflammation, altered myocardial energy metabolism, and ectopic fat accumulation. Advanced imaging studies, including cardiac magnetic resonance, demonstrate increased myocardial, epicardial, and pericardial fat content that is closely associated with visceral adiposity and hepatic steatosis [98]. In biopsy-proven MASLD, liver fibrosis severity correlates with LV hypertrophy, impaired myocardial deformation, and diastolic dysfunction, even after adjustment for traditional cardiometabolic risk factors, reinforcing the concept that fibrosis characterizes both hepatic and cardiovascular outcomes [99]. In addition, MASLD has been shown to be independently associated with an increased risk of major adverse cardiovascular events (MACE), including myocardial infarction, ischemic stroke, AF, and heart failure. Subclinical cardiac abnormalities—particularly LV diastolic dysfunction, impaired myocardial strain, left atrial remodeling, and increased arterial stiffness—are frequently detectable even in early disease stages (see Table 1). These findings underscore the systemic nature of MASLD and highlight the need for integrated cardiovascular risk assessment that incorporates liver disease stage, such as cACLD, and targeted cardiovascular imaging to optimize risk stratification and guide therapeutic strategies in clinical practice.

## 7. Non-Pharmacological Interventions: The Role of Lifestyle

Nutritional intervention is a cornerstone of MASLD treatment, exerting direct effects on the pathophysiological mechanisms driving hepatic steatosis and disease progression. The primary goal is the reduction in energy surplus and visceral adiposity: weight loss of at least 5% is associated with improvements in steatosis, 7–10% with benefits on inflammation and hepatocellular ballooning, and greater than 10% favoring a potential attenuation of fibrotic progression [100]. However, dietary quality plays a crucial role, even independently of weight loss. The Mediterranean diet is the most extensively studied dietary pattern in MASLD owing to its high content of fiber, monounsaturated fats, particularly from extra-virgin olive oil, polyunsaturated omega-3 fatty acids, fruits, vegetables, and whole grains, along with a reduced intake of simple sugars, ultra-processed foods, and saturated fats. Attention should be paid to limiting the consumption of added fructose and sugar-sweetened beverages, which are strongly implicated in de novo hepatic lipogenesis and IR [101,102].

Regular physical activity exerts beneficial effects on MASLD through mechanisms that are partly independent of weight loss. Aerobic exercise enhances fatty acid oxidation, reduces hepatic and visceral fat content, and improves insulin sensitivity, while resistance training contributes to preserving and increasing muscle mass, counteracting the sarcopenia which is frequently associated with MASLD and improving glucose metabolism [103]. Current guidelines recommend at least 150–300 min per week of moderate-intensity aerobic activity or 75–150 min of vigorous-intensity activity, combined with resistance exercises at least twice weekly [100]. Even gradual increases in daily physical activity and reductions in sedentary behavior are associated with clinical benefits. Within a personalized medicine framework, the integration of tailored physical activity programs alongside multidisciplinary support is a key strategy for improving adherence and long-term outcomes in the management of MASLD [104].

Beyond nutrition and physical activity, several additional lifestyle factors significantly influence the development and progression of MASLD by acting on metabolic, inflammatory, and neuroendocrine pathways. Sleep plays a central role in the regulation of energy homeostasis and insulin sensitivity: reduced sleep duration, increased sleep fragmentation, and the presence of disorders such as obstructive sleep apnea is associated with increased hepatic steatosis, systemic inflammation, and cardiovascular risk [105]. Therefore, improving sleep and the early identification of sleep–wake rhythm disorders become relevant therapeutic targets in patients with MASLD.

The management of psychological stress and psychosocial factors is another key component. Chronic stress and mood disorders may promote dysfunctional eating behaviors, reduce adherence to therapeutic interventions, and contribute to the activation of the hypothalamic–pituitary–adrenal axis with detrimental effects on glucose and lipid metabolism [106]. Psychological support combined with stress reduction techniques can help improve the long-term sustainability of lifestyle changes.

## 8. Pharmacological Approaches Targeting MASLD

Emerging pharmacotherapies for MASLD aim to address the underlying metabolic drivers of the disease, including IR, obesity, and dysregulated lipid metabolism. Among these, agents targeting incretin signaling and renal glucose handling have shown the most promise [107]. In fact, by improving glycemic control, promoting weight loss, reducing hepatic fat accumulation, and modulating inflammatory and fibrotic pathways, these therapies offer a physiologically targeted approach to slow or reverse disease progression [108]. Moreover, many of these drugs confer cardiovascular benefits, which is particularly relevant given the elevated risk of MACE in patients with MASLD. The following subsections detail the mechanisms and clinical impact of sodium–glucose cotransporter 2 (SGLT2) inhibitors, glucagon-like peptide-1 (GLP-1) receptor agonists, and dual glucose-dependent insulinotropic polypeptide (GIP)/GLP-1 receptor agonists in MASLD management (Table 2) [109,110].

### 8.1. Pleiotropic Effects of SGLT2 Inhibitors on MASLD and cACLD

SGLT2 inhibitors are a class of antidiabetic agents with pleiotropic effects extending to the improvement of hepatic steatosis and the modulation of MASLD progression. Their primary action mechanism involves the inhibition of renal glucose reabsorption leading to increased urinary glucose excretion and reduced plasma glucose levels. This effect decreases glucotoxicity, improves insulin sensitivity, and reduces intracellular lipid accumulation in the liver, counteracting a key driver of MASLD pathogenesis [124,125,126].

Beyond glucose-mediated effects, SGLT2 inhibitors promote modest weight loss, predominantly through the reduction in visceral adiposity. Decreased visceral fat results in lower FFA delivery to the liver, limiting lipotoxicity and attenuating hepatic inflammation [127]. Preclinical studies have demonstrated that these agents positively modulate inflammatory pathways and oxidative stress, reducing pro-inflammatory cytokine expression (TNF-α, IL-6) and improving hepatic mitochondrial function [111].

Numerous randomized clinical trials (RCTs) and observational studies have confirmed the beneficial effects of SGLT2 inhibitors in patients with MASLD and T2DM. Reported outcomes include significant reductions in hepatic steatosis as measured by quantitative imaging techniques (MRI—proton density fat fraction—PDFF), FibroScan-Controlled Attenuation Parameter (CAP), and improvements in liver function test biomarkers such as alanine-aminotransferase (ALT), aspartate aminotransferase (AST), and γ-glutamyl transpeptidase (γ-GT). Preliminary evidence also suggests a possible modulation of fibrotic pathways as indicated by improvements in non-invasive fibrosis markers, likely mediated through decreased oxidative stress and the inhibition of hepatic stellate cell activation [117].

Finally, SGLT2 inhibitors provide well-established cardiovascular and renal benefits, including reductions in blood pressure, improvements in lipid profiles, and lower risk of MACE. These effects are particularly relevant in patients with MASLD, who frequently present metabolic comorbidities [128,129]. This multimodal profile positions SGLT2 inhibitors as ideal candidates for integrated therapeutic strategies targeting MASLD within the broader context of metabolic disease management.

Dapagliflozin, an SGLT2 inhibitor, represents a promising pharmacological approach for the management of MASLD, particularly in patients with T2DM. RCTs have demonstrated that dapagliflozin significantly reduces hepatic fat content, improves liver enzyme levels, and modulates inflammatory markers. In a study by Shi et al., patients with T2DM and NAFLD/MASLD who were treated with dapagliflozin for 24 weeks showed a significant reduction in liver fat content and serum ALT, TNF-α, and IL-6 levels compared with controls [130]. Another randomized trial reported reductions in intrahepatic fat and visceral adiposity, together with improved glycemic control and decreased ALT levels after 12 weeks of treatment [131].

Consistent with these findings, a meta-analysis confirmed that dapagliflozin significantly reduces ALT, AST, and HOMA-IR while improving metabolic parameters such as BMI, triglycerides, and LDL cholesterol [132]. More recent prospective studies have also reported reductions in CAP scores and other steatosis markers, although direct effects on fibrosis were not consistently significant [117]. Preclinical evidence suggests that dapagliflozin enhances insulin sensitivity and hepatic fatty acid β-oxidation, thereby contributing to reduced hepatic lipid accumulation [133]. Overall, dapagliflozin shows a favorable profile for improving liver steatosis and metabolic parameters, supporting its potential therapeutic role in MASLD. However, its direct impact on fibrosis and long-term liver-related outcomes requires further investigation in larger, longer-duration randomized trials.

Empagliflozin, another SGLT2 inhibitor, has been extensively investigated for its potential hepatoprotective effects in MASLD, particularly in patients with T2DM. Clinical evidence from RCTs indicate that empagliflozin improves liver function test levels, reduces hepatic steatosis, and favorably modulates non-invasive fibrosis indices. In a phase II RCT involving patients with T2DM and MASLD, empagliflozin (10–25 mg daily) administered for 6 months significantly reduced serum ALT, AST, and γ-GT compared with standard care, while also decreasing the FIB-4 index and improving imaging-based steatosis grades assessed by ultrasound and magnetic resonance (MR), suggesting benefits for both steatosis and surrogate fibrosis markers [134,135].

In a prospective trial comparing empagliflozin with pioglitazone in patients with MASLD and T2DM, empagliflozin was associated with improvements in liver steatosis assessed by CAP and reductions in liver stiffness, alongside significant decreases in body weight and visceral fat, despite similar changes in conventional biochemical indices between treatment groups [136]. Empagliflozin has also been shown to reduce intrahepatic lipid content more effectively than sitagliptin in patients with early T2DM and MASLD and to improve hepatic insulin sensitivity, highlighting its beneficial effects on ectopic fat accumulation beyond glycemic control [136,137]. Meta-analytical evidence further supports the hepatoprotective profile of empagliflozin in NAFLD/MASLD, with pooled data demonstrating significant reductions in ALT, AST, and γ-GT, although heterogeneity among studies limits definitive conclusions regarding long-term outcomes and the impact on liver fibrosis [138].

Mechanistic insights from preclinical models corroborate these clinical observations, showing that empagliflozin attenuates hepatic lipid accumulation, suppresses inflammatory signaling, and downregulates profibrotic gene expression, with effects that may be partially independent of weight loss and glycemic improvement [137]. Together, these findings suggest that empagliflozin holds promise as a metabolic therapeutic agent in MASLD, although larger and longer-term randomized trials with histological endpoints are required to confirm its effects on fibrosis progression and clinically meaningful liver outcomes.

Ertugliflozin, an SGLT2 inhibitor, has also demonstrated encouraging effects in the management of MASLD. Clinical evidence suggests that ertugliflozin treatment leads to significant reductions in liver steatosis assessed by ultrasound, and improvements in liver enzyme profiles, such as ALT and AST, compared with placebo or standard therapies after approximately 24 weeks of treatment [139]. Pooled analyses from the phase 3 VERTIS program further demonstrated that ertugliflozin consistently reduced serum ALT and AST levels over 52 weeks, with greater reductions observed in patients with higher baseline transaminase levels. These changes were moderately correlated with improvements in glycemic control and body weight, suggesting an interaction between metabolic and hepatic effects [140]. Additional studies in MASLD populations indicate that ertugliflozin, either as a monotherapy or in combination with other agents such as vitamin E, improves metabolic parameters, enhances insulin sensitivity, and reduces non-invasive biomarkers of liver fibrosis such as FIB-4, supporting its potential as a therapeutic strategy targeting both metabolic dysfunction and liver disease in patients with MASLD and T2DM [141].

Canagliflozin is primarily a highly selective SGLT2 inhibitor, but it also exerts a modest inhibitory effect on SGLT1, classifying it as a dual SGLT1/SGLT2 inhibitor. Growing evidence suggests it has beneficial effects on MASLD. In a six-month prospective study involving patients with T2DM and NAFLD/MASLD, treatment with canagliflozin (100 mg/day) resulted in significant reductions in liver steatosis, assessed by MRI-PDFF, along with decreases in body weight, HbA1c, HOMA-IR, and inflammatory markers such as hs-CRP [142]. Another prospective cohort study including 35 patients with NAFLD/MASLD treated with canagliflozin reported significant reductions in liver enzymes (AST, ALT, γ-GT), triglyceride levels, and the FIB-4 index, as well as body weight after 3 and 6 months of treatment, indicating improvements in both liver function and the metabolic parameters associated with steatosis [143].

Furthermore, meta-analyses evaluating SGLT2 inhibitors, including canagliflozin, have shown consistent reductions in liver enzyme levels and improvements in selected markers of hepatic steatosis compared with control therapies in patients with T2DM and NAFLD/MASLD, supporting a class-related hepatoprotective effect [144]. Preclinical studies reinforce these findings, demonstrating that canagliflozin modulates hepatic lipid metabolism and inflammatory pathways, reduces oxidative stress, and improves mitochondrial function in animal models of MASLD [145].

Although most available evidence concerns patients with MASLD without advanced fibrosis, SGLT2 inhibitors have been increasingly investigated for their potential relevance in cACLD. In this setting their effects may be extended beyond steatosis reduction, potentially involving mechanisms related to fibrogenesis, intrahepatic vascular resistance, and systemic hemodynamic modulation, though definitive results are still lacking [146,147].

By improving IR, reducing visceral adiposity, and attenuating chronic low-grade inflammation, SGLT2 inhibitors may indirectly counteract HSC activation and fibrotic progression. Preclinical data demonstrate the reduced expression of profibrotic mediators and oxidative stress markers, while clinical studies report improvements in non-invasive fibrosis indices and liver stiffness measurements [117,148,149]. However, these findings are primarily derived from indirect or surrogate markers, and their clinical significance in cACLD remains to be fully established. Moreover, their natriuretic and osmotic diuretic properties may contribute to the modulation of the hyperdynamic circulation typical of advanced chronic liver disease. Improvements in endothelial function and reductions in oxidative stress could theoretically influence intrahepatic vascular tone and portal pressure, although direct hemodynamic evidence remains limited [150,151].

Given the high burden of cardiovascular comorbidities in patients with cACLD, the well-established cardioprotective effects of SGLT2 inhibitors—including reductions in heart failure hospitalization and improvements in ventricular remodeling—are particularly relevant in this population [152,153]. Overall, while current data suggests a favorable safety profile in compensated liver disease [154], dedicated randomized trials in patients with advanced fibrosis and compensated cirrhosis are still needed in order to clarify long-term hepatic outcomes and their impact on clinical endpoints, including evolution to decompensation. Moreover, heterogeneity across study designs and populations contributes to variability in reported outcomes, and some studies have failed to demonstrate significant improvements in fibrosis-related endpoints despite showing metabolic benefits.

### 8.2. Impact of GLP-1 Receptor Agonists on MASLD and cACLD

GLP-1 receptor agonists represent one of the most promising therapeutic classes for MASLD due to their potent effects on body weight, insulin sensitivity, and systemic metabolic regulation [155]. These agents enhance glucose-dependent insulin secretion, suppress inappropriate glucagon release, delay gastric emptying, and reduce appetite through central nervous system mechanisms, resulting in substantial and sustained weight loss [156]. Given the strong association between excess adiposity—particularly visceral fat—and liver steatosis, weight reduction is a key mediator of the beneficial effects observed with GLP-1 receptor agonists.

Beyond weight loss, GLP-1 receptor agonists exert direct and indirect effects on hepatic metabolism. Improved insulin sensitivity reduces hepatic DNL and limits FFA flux from adipose tissue to the liver, thereby decreasing intrahepatic triglyceride accumulation [112]. Experimental data suggest that GLP-1 signaling may also directly modulate hepatocellular lipid metabolism and mitochondrial function, leading to reduced oxidative stress and improved cellular homeostasis [116]. These mechanisms together translate into reductions in hepatic fat content, as consistently demonstrated by imaging-based studies using MRI–PDFF and CAP [120].

Clinical trials in patients with MASLD and T2DM, as well as in individuals without diabetes, have shown significant improvements in liver enzymes and necro-inflammatory activity following treatment with GLP-1 receptor agonists [120]. Notably, histological studies have reported higher rates of steatohepatitis resolution compared with placebo, accompanied by reductions in hepatocellular ballooning and lobular inflammation [122]. Although improvements in fibrosis stage appear more modest and may require longer treatment durations, reductions in non-invasive fibrosis markers and liver stiffness suggest a possible modulation of fibrotic pathways, as reflected by surrogate markers. However, several randomized trials and meta-analyses have reported only modest or non-significant improvements in fibrosis stage, suggesting that antifibrotic effects may require longer treatment duration or may be limited to specific subgroups [157,158].

In addition to their hepatic benefits, GLP-1 receptor agonists confer well-established cardiovascular protection, including reductions in MACE, improvements in blood pressure and lipid profiles, and favorable effects on endothelial function [159]. These pleiotropic benefits are particularly relevant in MASLD where CVD is the leading cause of mortality. Overall, GLP-1 receptor agonists target multiple pathogenic drivers of MASLD—obesity, IR, inflammation, and cardiometabolic risk—supporting their role as disease-modifying agents within an integrated therapeutic strategy [160,161].

Liraglutide, a GLP-1 receptor agonist, is one of the first incretin-based therapies to be investigated in MASLD and its progressive form, metabolic dysfunction-associated steatohepatitis (MASH). In the Liraglutide Efficacy and Action in NASH (LEAN) trial, liraglutide administered at 1.8 mg daily for 48 weeks demonstrated a higher rate of histological resolution of steatohepatitis without worsening of fibrosis compared with placebo, and improvements in surrogate biochemical markers of liver injury such as ALT and γ-GT were also observed [160]. These findings suggest that liraglutide may attenuate hepatic steatosis and inflammation, likely mediated through weight reduction and enhanced insulin sensitivity [161], although its impact on fibrotic pathways remains less well established and underscore the need for larger, longer RCTs with histological endpoints.

Evidence on dulaglutide in MASLD/NAFLD, although less extensive than for other GLP-1 receptor agonists, suggests promising metabolic and hepatic benefits. In the D-LIFT RCT, weekly dulaglutide significantly reduced hepatic fat content, as measured by MRI-PDFF, and improved markers of liver injury such as γ-GT in patients with T2DM and NAFLD/MASLD [140]. Similar findings were observed in a two-center open-label trial in which dulaglutide led to reductions in liver fat and improvements in glycemic control and body weight [162]. Mechanistic studies also suggest that dulaglutide may attenuate hepatic steatosis via weight-independent pathways, including the modulation of lipid metabolism and hepatic inflammation [163]. Meta-analyses of GLP-1 receptor agonists confirm that the class, including dulaglutide, reduces liver fat content and improves liver enzymes in MASLD/NAFLD, although the volume of high-quality evidence specifically for dulaglutide remains smaller than that for liraglutide and semaglutide [164,165]. Overall, these data support dulaglutide as a potential therapeutic option for hepatic steatosis in patients with metabolic disease, but larger, long-term studies are required to establish its effects on fibrosis and histological endpoints.

Semaglutide, a long-acting GLP-1 receptor agonist originally developed for T2DM and later approved at higher doses for obesity management, has emerged as a promising therapeutic option in MASLD and MASH. Several RCTs and pooled analyses demonstrate that semaglutide significantly improves key histological and clinical outcomes of liver disease. A recent large meta-analysis of 22 RCTs showed that semaglutide nearly doubled the likelihood of histological resolution of MASH compared with control treatments, substantially reduced hepatic steatosis as measured by percentage of liver fat, and improved non-invasive markers of fibrosis such as the Enhanced Liver Fibrosis (ELF) score [166]. However, evidence for consistent effects on hepatic fibrosis remains limited across studies. Complementary systematic reviews focusing on MASLD/MASH populations corroborate these findings, showing that semaglutide increases the odds of NASH/MASH resolution and improvement in individual histological features such as steatosis, hepatocellular ballooning, and lobular inflammation, and reduces liver stiffness assessed by elastography. Despite these benefits, semaglutide’s impact on fibrosis stage remains variable, particularly in shorter-term studies [166,167]. Across clinical contexts, semaglutide treatment is consistently associated with significant reductions in serum liver enzymes including ALT and AST, improvements in glycemic control and body weight, and enhanced cardiometabolic parameters that are closely linked to MASLD pathogenesis [167].

Real-world cohort analyses further support semaglutide’s beneficial liver outcomes: in patients with NAFLD and T2DM, semaglutide use was associated with a lower risk of major adverse liver outcomes (MALO) and all causes of mortality compared with other glucose-lowering drugs such as SGLT2 inhibitors, DPP-4 inhibitors, and thiazolidinediones [168]. Mechanistic insights from imaging and metabolic studies suggest that semaglutide may reduce hepatic steatosis not only through weight loss and improved insulin sensitivity, but also via the suppression of DNL and favorable shifts in lipid handling within the liver [169].

Large phase 3 trials such as the ESSENCE program have reported that once-weekly semaglutide at 2.4 mg leads to the resolution of steatohepatitis without worsening of fibrosis in a majority of treated patients compared with placebo, alongside substantial mean weight loss (approximately 10.5%) and improvements in metabolic biomarkers, leading to regulatory guidance updates that support semaglutide use in MASH with moderate-to-advanced fibrosis [170]. Taken together, these data position semaglutide as a cornerstone of metabolic pharmacotherapy for MASLD/MASH, although longer-term studies and analyses of fibrosis endpoints remain key areas of ongoing investigation.

Based on its metabolic, anti-steatotic, and anti-inflammatory effects on MASLD/MASH, interest has been emerging regarding the potential role of GLP-1 receptor agonists in patients with cACLD. GLP-1 receptor agonists have been shown to reduce liver fat content and improve histological features of steatohepatitis, including hepatocellular ballooning and lobular inflammation, without worsening fibrosis in MASLD/MASH populations [171]. In more advanced stages of liver disease, therapeutic goals would theoretically extend beyond the resolution of steatohepatitis to include fibrosis stabilization, the delay of portal hypertension progression and the reduction in hepatic decompensation risk. Real-world evidence suggests that the initiation of GLP-1 receptor agonists may be associated with improved long-term liver-related outcomes, including hepatic decompensation, clinically significant portal hypertension, hepatocellular carcinoma, and liver transplantation in patients with MASLD cirrhosis and T2DM [172]. Nevertheless, these findings are observational in nature and should be interpreted with caution due to potential residual confounding. Emerging comparative studies, particularly involving semaglutide, indicate possible advantages over other agents within the class in terms of mortality and composite liver outcome, conferring superior clinical benefits in the reduction in all causes of mortality and MALO compared to other agents within the class [173]. These effects are likely mediated through weight reduction, improved metabolic control, and the attenuation of systemic inflammation, all of which are key drivers of fibrogenesis and portal hypertension progression. Reductions in liver stiffness assessed by non-invasive methods further suggest a potential effect on the fibrotic burden; however, histological evidence in advanced disease stages remains limited [171,174]. The established cardiovascular benefits of GLP-1 receptor agonists are particularly relevant in cACLD, where cardiometabolic comorbidities substantially influence prognosis and mortality [118]. However, safety considerations remain crucial in patients with advanced liver disease, frailty, or prior decompensation, underscoring the need for careful patient selection. Overall, while GLP-1 receptor agonists appear promising across the MASLD spectrum, their specific role in cACLD remains to be defined, and dedicated prospective studies are required to establish their efficacy and safety in this setting.

### 8.3. A Novel Therapeutic Approach to MASLD and cACLD: Dual GLP-1/GIP Receptor Agonists

Dual agonists targeting both GLP-1 and GIP receptors are an emerging class of therapeutics with substantial potential in the management of MASLD [110]. These agents leverage the complementary mechanisms of GLP-1 and GIP signaling to achieve superior metabolic outcomes compared to GLP-1 receptor agonist monotherapy. Dual agonism enhances glucose-dependent insulin secretion and suppresses inappropriate glucagon release, improving glycemic control while simultaneously promoting greater weight loss through central appetite regulation and delayed gastric emptying [113,114].

Beyond glycemic effects, dual GIP/GLP-1 receptor agonists exert significant effects on adipose tissue and hepatic metabolism. They modulate adipocyte function, increasing lipid oxidation and reducing ectopic lipid deposition in the liver, thereby attenuating hepatic steatosis [115]. Experimental studies suggest that these agents also improve mitochondrial function and reduce oxidative stress in hepatocytes, mitigating lipotoxic injury and inflammation [119]. Early-phase clinical trials have demonstrated that dual agonists lead to more pronounced reductions in liver fat content—assessed via MRI-PDFF and other imaging modalities—compared with GLP-1 monotherapy, and they are associated with improvements in liver enzyme levels and necro-inflammatory activity [120].

Importantly, dual agonists may offer indirect antifibrotic benefits. By reducing hepatic fat and systemic inflammation, and by improving insulin sensitivity, these agents could potentially slow the progression of fibrosis, although long-term histological data are still limited [121]. Robust metabolic improvements, including reductions in body weight, visceral adiposity, and cardiovascular risk factors, underscore their potential as a disease-modifying strategy for patients with MASLD, particularly those with T2DM or obesity-related comorbidities [113].

It is important to emphasize that the potential role of dual GIP/GLP-1 receptor agonists may extend beyond the early stages of MASLD to patients with cACLD, although evidence in this setting remains limited. In this population, therapeutic priorities would include fibrosis stabilization, slowing the progression of portal hypertension, and reducing the risk of hepatic decompensation [123].

The marked and sustained weight loss observed with dual agonists—generally greater than that achieved with GLP-1 receptor agonists alone—may be particularly relevant in metabolically driven advanced fibrosis, given the central role of visceral adiposity and IR in fibrogenesis and portal pressure dynamics [123,175]. Emerging imaging-based analyses report reductions in liver stiffness and improvements in non-invasive fibrosis markers following treatment with dual incretin agonists; however, these findings are primarily based on surrogate endpoints, and definitive histological evidence in patients with advanced fibrosis or compensated cirrhosis is still lacking. Overall, although dual GIP/GLP-1 receptor agonists represent a promising therapeutic option across the MASLD spectrum, their potential role in cACLD remains speculative, and long-term studies specifically evaluating their safety and efficacy in compensated and decompensated cACLD are still required.

### 8.4. Safety, Tolerability, and Clinical Considerations of Emerging Therapies

Although the pharmacological agents discussed show promising metabolic and hepatic benefits, their use in clinical practice requires careful considerations regarding safety, tolerability, and patient selection. Adverse gastrointestinal effects, such as nausea, vomiting, and constipation, particularly with GLP-1 receptor agonists and dual agonists, may limit tolerability in some patients, especially during treatment initiation [176,177,178]. Although GLP-1 receptor agonists are not formally contraindicated in patients with chronic liver disease according to current product labeling, clinical experience in advanced hepatic impairment and cirrhosis remains limited, and their use should be approached with caution. In patients with cACLD, caution is warranted due to potential issues such as sarcopenia and frailty. In patients with MASLD and cACLD, sarcopenia is highly prevalent and represents a major prognostic factor for mortality and decompensation. GLP-1 receptor agonists induce significant weight loss, which may include a reduction in lean body mass. Although generally beneficial in metabolic terms, this effect warrants caution in advanced liver disease in which the preservation of muscle mass is crucial, and it should be mitigated through nutritional optimization and resistance exercise strategies. Although current data suggest an overall favorable safety profile in compensated disease, evidence in decompensated cirrhosis remains limited, and these agents should be used with careful clinical monitoring [179,180,181].

SGLT2 inhibitors are generally well tolerated but may be associated with an increased risk of genitourinary infections and volume depletion, which may be clinically relevant in patients with decompensated advanced liver disease [182,183,184].

Thus, treatment decisions should be individualized, considering metabolic profile, liver disease stage, cardiovascular comorbidities, and overall sarcopenia, ideally within a multidisciplinary framework.

## 9. Conclusions

MASLD is one of the major hepatic consequences of obesity and metabolic dysfunction which has been emerging as a systemic disease with significant cardiovascular and metabolic implications. Advances in our understanding of the underlying pathophysiological mechanisms—ranging from IR to gut dysbiosis, and from chronic inflammation to oxidative stress—have enabled the development of targeted therapeutic strategies. In this context, metabolically active agents such as SGLT2 inhibitors, GLP-1 receptor agonists, and dual GIP/GLP-1 receptor agonists have demonstrated consistent benefits on body weight, insulin sensitivity, hepatic steatosis, and non-invasive markers of fibrosis. Although the current evidence is encouraging, especially for semaglutide, further long-term studies are needed to clarify their impact on progression to MASLD, advanced fibrosis, and clinical outcomes, especially in advanced stages of disease.

Future research should focus on long-term studies assessing hard clinical outcomes, including the prevention of hepatic decompensation, progression of portal hypertension, incidence of hepatocellular carcinoma, and liver-related mortality, particularly in patients with cACLD. While current evidence supports improvements in metabolic parameters, hepatic steatosis, and non-invasive fibrosis markers, robust data on clinical hepatic outcomes remain limited. Filling this gap is essential in order to determine whether emerging pharmacological therapies can be fully integrated into the standard of care for advanced stages of MASLD.

Although both GLP-1 receptor agonists and SGLT2 inhibitors consistently reduce MACE in diabetic and high-risk populations, evidence specifically in MASLD cohorts remains largely indirect, and the independent contribution of hepatic improvement to cardiovascular risk reduction remains to be defined. Overall, future research will need to clarify whether metabolic and histological improvements can translate into significant reductions in morbidity and mortality related to chronic liver disease. Such research will be crucial to defining the disease-modifying role of emerging therapeutic strategies in MASLD.

## Figures and Tables

**Table 1:** Pathophysiological mechanisms, clinical manifestations, and diagnostic markers and techniques of cardio and cerebrovascular system involvement in MASLD.

	Pathophysiological Mechanisms	Clinical Manifestations	Diagnostic Markers and Techniques
**Peripheral Vascular** **System**	Oxidative stress, pro-thrombotic state, platelet activation, RAAS activation [71,74].	Endothelial dysfunction, accelerated atherosclerosis, increased arterial stiffness, hypertension.	Increased carotid (IMT) by Echo-Color Doppler, carotid plaque, impaired FMD.
**Cardiac Structure**	Chronic inflammation, ectopic fat accumulation, myocardial lipotoxicity [75,76,77].	Left ventricular (LV) remodeling, concentric LV hypertrophy, left atrial enlargement.	Cardiac hypertrophy by echocardiography, cardiac MRI (epicardial/pericardial fat accumulation).
**Cardiac Function**	Insulin resistance, myocardial fibrosis, altered myocardial energy metabolism [67,78,80].	Diastolic dysfunction (frequent even with preserved ejection fraction), subclinical systolic impairment.	Reduced (GLS) via Speckle-tracking, Tissue Doppler Imaging (E’ velocity).
**Cardiac Rhythm**	Atrial structural remodeling, autonomic imbalance, atrial fibrogenesis driven by cytokines (TNF-α, IL-6) [92,93,94].	Atrial fibrillation (AF), arrhythmias.	ECG, 24 h Holter ECG, clinical monitoring.
**Cerebrovascular System**	Pro-thrombotic state, embolism (AF-dependent), accelerated carotid atherosclerosis [96,97].	Ischemic stroke, Cerebro-Vascular Chronic Disease (CVCD).	MACE risk assessment, carotid screening.
**Systemic Inflammation**	Dysregulated hepatokine and adipokine [95].	Chronic low-grade inflammation, dyslipidemia.	Increased levels of: CRP, Fetuin-A, Leptin, TNF-α, IL-6, total cholesterol and LDL, triglycerides.

**Table 2:** Mechanisms and clinical impact of SGLT2 inhibitors, GLP-1 receptor agonists, and dual GIP/GLP-1 receptor agonists in MASLD management.

SGLT2-i	GLP-1 RA	GIP/GLP-1 RA
↓ body weight [111]	↓ body weight [112]	↓ body weight [113,114]
↓ pro-inflammatory cytokine expression [111]	↑ insulin sensitivity [112]	↑ lipid oxidation [115]
↑ hepatic mitochondrial function [111]	↓ de novo lipogenesis [116]	↓ ectopic lipid deposition [115]
↓ AST/ALT hepatic steatosis [117]	↓ intrahepatic triglyceride accumulation [116]	↓ lipotoxic injury, hepatic steatosis and inflammation [115]
↓ hepatic fibrosis and steatosis [117]	↓ oxidative stress [116]	↓ AST/ALT, liver fat content, and necro-inflammatory activity [118,119]
↓ HOMA-IR [117]	↓ AST and ALT and liver fibrosis [120]	↓ hepatic fibrosis [121]
↓ FIB-4 index [111]	↓ hepatocellular ballooning and lobular inflammation [120,122]	↓ progression of portal hypertension and hepatic decompensation [113,123]

## Data Availability

No new data were created or analyzed in this study.

## References

[1] Li Y., Yang P., Ye J., Xu Q., Wu J., Wang Y. (2024). Updated mechanisms of MASLD pathogenesis. Lipids Health Dis..

[2] Li M., Xie W. (2024). Are there all-cause mortality differences between metabolic dysfunction-associated steatotic liver disease subtypes?. J. Hepatol..

[3] Le M.H., Yeo Y.H., Li X., Li J., Zou B., Wu Y., Ye Q., Huang D.Q., Zhao C., Zhang J. (2022). Global NAFLD prevalence: A systematic review and meta-analysis. Clin. Gastroenterol. Hepatol..

[4] Younossi Z.M., Golabi P., Paik J.M., Henry A., Van Dongen C., Henry L. (2023). The global epidemiology of nonalcoholic fatty liver disease (NAFLD) and nonalcoholic steatohepatitis (NASH): A systematic review. Hepatology.

[5] Magliano D.J., Boyko E.J., Genitsaridi I., Piemonte L., Riley P., Salpea P., International Diabetes Federation (2025). IDF Diabetes Atlas.

[6] Zhang X., Linden S., Levesley C.R., He X., Yang Z., Barnet S.D., Cheung R., Ji F., Nguyen M.H. (2025). Projected Trends in Meta-bolic Dysfunction-Associated Steatotic Liver Disease Mortality Through 2040. JAMA Netw. Open.

[7] Targher G., Valenti L., Byrne C.D. (2025). Metabolic Dysfunction-Associated Steatotic Liver Disease. N. Engl. J. Med..

[8] Licata A., Russo G.T., Giandalia A., Cammilleri M., Asero C., Cacciola I. (2023). Impact of sex and gender on clinical management of patients with advanced chronic liver disease and type 2 diabetes. J. Pers. Med..

[9] Joo S.K., Kim W. (2024). Sex differences in metabolic dysfunction-associated steatotic liver disease: A narrative review. Ewha Med. J..

[10] Fabbrini E., Sullivan S., Klein S. (2010). Obesity and nonalcoholic fatty liver disease: Biochemical, metabolic, and clinical implications. Hepatology.

[11] Cusi K. (2009). Role of insulin resistance and lipotoxicity in non-alcoholic steatohepatitis. Clin. Liver Dis..

[12] Tilg H., Moschen A.R. (2010). Evolution of inflammation in nonalcoholic fatty liver disease: The multiple parallel hits hypothesis. Hepatology.

[13] Kalavalapalli S., Leiva E.G., Lomonaco R., Chi X., Shrestha S., Dillard R., Budd J., Romero J.P., Li C., Bril F. (2023). Adipose Tissue Insulin Resistance Predicts the Severity of Liver Fibrosis in Patients With Type 2 Diabetes and NAFLD. J. Clin. Endocrinol. Metab..

[14] Bril F., Cusi K. (2017). Liver fat accumulation as a barometer of insulin responsiveness again points to adipose tissue as the culprit. Hepatology.

[15] Donnelly K.L., Smith C.I., Schwarzenberg S.J., Jessurun J., Boldt M.D., Parks E.J. (2005). Sources of fatty acids stored in liver and secreted via lipoproteins in patients with NAFLD. J. Clin. Investig..

[16] Lambert J.E., Ramos-Román M.A., Browning J.D., Parks E.J. (2014). Increased de novo lipogenesis is a hallmark of NAFLD in humans. Gastroenterology.

[17] Petersen K.F., Dufour S., Befroy D., Lehrke M., Hendler R.E., Shulman G.I. (2005). Reversal of nonalcoholic hepatic steatosis, hepatic insulin resistance, and hyperglycemia by moderate weight reduction in patients with type 2 diabetes. Diabetes.

[18] Schwarz J.M., Neese R.A., Turner S., Dare D., Hellerstein M.K. (1995). Short-term alterations in carbohydrate energy intake in humans. Striking effects on hepatic glucose production, de novo lipogenesis, lipolysis, and whole-body fuel selection. J. Clin. Investig..

[19] Fabbrini E., Mohammed B.S., Magkos F., Korenblat K.M., Patterson B.W., Klein S. (2008). Alterations in adipose tissue and hepatic lipid kinetics in obese men and women with nonalcoholic fatty liver disease. Gastroenterology.

[20] Adiels M., Taskinen M.R., Packard C., Caslake M.J., Soro-Paavonen A., Westerbacka J., Vehkavaara S., Häkkinen A., Olofsson S.O., Yki-Järvinen H. (2006). Overproduction of large VLDL particles is driven by increased liver fat content in man. Diabetologia.

[21] Kumashiro N., Erion D.M., Zhang D., Kahn M., Beddow S.A., Chu X., Still C.D., Gerhard G.S., Han X., Dziura J. (2011). Cellular mechanisms of hepatic insulin resistance in humans with NAFLD. Proc. Natl. Acad. Sci. USA.

[22] Chaurasia B., Ying L., Talbot C.L., Maschek J.A., Cox J., Schuchman E.H., Hirabayashi Y., Holland W.L., Summers S.A. (2021). Ceramides are necessary and sufficient for diet-induced impairment of thermogenic adipocytes. Mol. Metab..

[23] Sanyal A.J., Campbell-Sargent C., Mirshahi F., Rizzo W.B., Contos M.J., Sterling R.K., Luketic V.A., Shiffman M.L., Clore J.N. (2001). Nonalcoholic steatohepatitis: Association of insulin resistance and mitochondrial abnormalities. Gastroenterology.

[24] Videla L.A., Rodrigo R., Orellana M., Fernandez V., Tapia G., Quiñones L., Varela N., Contreras J., Lazarte R., Csendes A. (2004). Oxidative stress-related parameters in the liver of non-alcoholic fatty liver disease patients. Clin. Sci..

[25] Serviddio G., Bellanti F., Tamborra R., Rollo T., Capitanio N., Romano A.D., Sastre J., Vendemiale G., Altomare E. (2008). Uncoupling protein-2 (UCP2) induces mitochondrial proton leak and increases susceptibility of non-alcoholic ste-atohepatitis (NASH) liver to ischaemia-reperfusion injury. Gut.

[26] Liu W., Baker S.S., Baker R.D., Zhu L. (2015). Antioxidant Mechanisms in Nonalcoholic Fatty Liver Disease. Curr. Drug Targets.

[27] Puri P., Mirshahi F., Cheung O., Natarajan R., Maher J.W., Kellum J.M., Sanyal A. (2008). J Activation and dysregulation of the unfolded protein response in nonalcoholic fatty liver disease. Gastroenterology.

[28] Lake A.D., Novak P., Hardwick R.N., Flores-Keown B., Zhao F., Klimecki W.T., Cherrington N.J. (2014). The adaptive endoplasmic reticulum stress response to lipotoxicity in progressive human nonalcoholic fatty liver disease. Toxicol. Sci..

[29] Verdam F.J., Greve J.W., Roosta S., van Eijk H., Bouvy N., Buurman W.A., Rensen S.S. (2011). Small intestinal alterations in se-verely obese hyperglycemic subjects. J. Clin. Endocrinol. Metab..

[30] Wree A., Eguchi A., McGeough M.D., Peña C.A., Johnson C.D., Canbay A., Hoffman H.M., Feldstein A.E. (2014). NLRP3 inflammasome activation results in hepatocyte pyroptosis, liver inflammation, and fibrosis in mice. Hepa-Tology.

[31] Mridha A.R., Wree A., Robertson A.A.B., Yeh M.M., Johnson C.D., Van Rooyen D.M., Haczeyni F., Teoh N.C.-H., Savard C., Ioannou G.N. (2017). NLRP3 inflammasome blockade reduces liver inflammation and fibrosis in experimental NASH in mice. J. Hepatol..

[32] Zhu L., Baker S.S., Gill C., Liu W., Alkhouri R., Baker R.D., Gill S.R. (2013). Gut microbiota and endotoxemia in NAFLD patients. Hepatology.

[33] Boursier J., Mueller O., Barret M., Machado M., Fizanne L., Araujo-Perez F., Guy C.D., Seed P.C., Rawls J.F., David L.A. (2016). Gut microbiota and liver fibrosis in NAFLD: The severity of nonalcoholic fatty liver disease is associated with gut dysbiosis and shift in the metabolic function of the gut microbiota. Hepatology.

[34] Zhu S., Zou M., Wu Q., Zou Y., Tan T., Huang Z., Gong Z., Luo H., Dong X. (2026). The Gut-Liver Axis in Metabolic Dysfunction-Associated Steatotic Liver Disease: From Mechanistic Insights to Precision Therapeutics. FASEB J..

[35] Hoyles L., Fernández-Real J.M., Federici M., Serino M., Abbott J., Charpentier J., Heymes C., Luque J.L., Anthony E., Barton R.H. (2018). Molecular phenomics and metagenomics of hepatic steatosis in non-diabetic obese women. Nat. Med..

[36] Pipitone R.M., Ciccioli C., Infantino G., La Mantia C., Parisi S., Tulone A., Pennisi G., Grimaudo S., Petta S. (2023). MAFLD: A multisystem disease. Ther. Adv. Endocrinol. Metab..

[37] Younossi Z.M., Kalligeros M., Henry L. (2025). Epidemiology of metabolic dysfunction-associated steatotic liver disease. Clin. Mol. Hepatol..

[38] Huh Y., Cho Y.J., Nam G.E. (2022). Recent epidemiology and risk factors of nonalcoholic fatty liver disease. J. Obes. Metab. Syndr..

[39] Koliaki C., Dalamaga M., Kakounis K., Liatis S. (2025). Metabolically healthy obesity and metabolic dysfunction-associated steatotic liver disease (MASLD): Navigating the controversies in disease development and progression. Curr. Obes. Rep..

[40] Tsochatzis E., Papatheodoridis G.V., Hadziyannis E., Georgiou A., Kafiri G., Tiniakos D.G., Manesis E.K., Archimandritis A.J. (2008). Serum adipokine levels in chronic liver diseases: Association of resistin levels with fibrosis severity. Scand. J. Gastroenterol..

[41] Crişan D., Avram L., Morariu-Barb A., Grapa C., Hirişcau I., Crăciun R., Donca V., Nemeş A. (2025). Sarcopenia in MASLD—Eat to beat steatosis, move to prove strength. Nutrients.

[42] Wang P., Liu X., Du X., Qiu L., Liu Y., Xu S., Zhang Y., Zhang J. (2025). Prevalence and risk of metabolic dysfunction-associated steatotic liver disease in patients with sarcopenic obesity: A systematic review and meta-analysis. Nutr. Metab..

[43] Polyzos S.A., Vachliotis I.D., Mantzoros C.S. (2023). Sarcopenia, sarcopenic obesity and nonalcoholic fatty liver disease. Metabolism.

[44] Omana-Guzman I., Rosas-Diaz M., Martinez-Lopez Y.E., Perez-Navarro L.M., Diaz-Badillo A., Alanis A., Bustamante A., Castillo-Ruiz O., del Toro-Cisneros N., Esquivel-Hernandez D.A. (2024). Strategic interventions in clinical randomized trials for metabolic dysfunction-associated steatotic liver disease (MASLD) and obesity in the pediatric population: A systematic review with meta-analysis and bibliometric analysis. BMC Med..

[45] Ducluzeau P.H., Boursier J., Bertrais S., Dubois S., Gauthier A., Rohmer V., Gagnadoux F., Lefthériotis G., Calès P., Andriantsitohaina R. (2013). MRI measurement of liver fat content predicts the metabolic syndrome. Diabetes Metab..

[46] Guo Z., Zhou L., Jensen M.D. (2006). Acute hyperinsulinemia inhibits intramyocellular triglyceride synthesis in high-fat-fed obese rats. J. Lipid Res..

[47] Shanka N.Y., Pavlov C.S., Mekonnen N.L. (2025). Non-invasive methods for diagnosing portal hypertension and variceal bleeding due to liver cirrhosis secondary to NAFLD/MASLD: Systematic review. Front. Med..

[48] Madir A., Grgurevic I., Tsochatzis E.A., Pinzani M. (2024). Portal hypertension in patients with nonalcoholic fatty liver disease: Current knowledge and challenges. World J. Gastroenterol..

[49] Mitten E.K., Portincasa P., Baffy G. (2023). Portal hypertension in nonalcoholic fatty liver disease: Challenges and paradigms. J. Clin. Transl. Hepatol..

[50] Nababan S.H.H., Lesmana C.R.A. (2022). Portal hypertension in nonalcoholic fatty liver disease: From pathogenesis to clinical practice. J. Clin. Transl. Hepatol..

[51] López-Méndez I., Juárez-Hernández E., Soriano-Márquez J.P., Uribe M., Castro-Narro G. (2025). Early portal hypertension in metabolic dysfunction-associated steatotic liver disease: A concise review. Expert. Rev. Gastroenterol. Hepatol..

[52] Baffy G. (2018). Origins of portal hypertension in nonalcoholic fatty liver disease. Dig. Dis. Sci..

[53] Theofilis P., Vordoni A., Nakas N., Kalaitzidis R.G. (2022). Endothelial dysfunction in nonalcoholic fatty liver disease: A systematic review and meta-analysis. Life.

[54] Argo C.K., Northup P.G., Al-Osaimi A.M., Caldwell S.H. (2009). Systematic review of risk factors for fibrosis progression in non-alcoholic steatohepatitis. J. Hepatol..

[55] Garcia-Tsao G., Sanyal A.J., Grace N.D., Carey W. (2007). Prevention and management of gastroesophageal varices and variceal hemorrhage in cirrhosis. Hepatology.

[56] Bosch J., Abraldes J.G., Groszmann R. (2003). Current management of portal hypertension. J. Hepatol..

[57] Tripathi D., Stanley A.J., Hayes P.C., Travis S., Armstrong M.J., Tsochatzis E.A., Rowe I.A., Roslund N., Ireland H., Lomax M. (2020). Transjugular intrahepatic portosystemic stent-shunt in the management of portal hypertension. Gut.

[58] Hirooka M., Koizumi Y., Miyake T., Ochi H., Tokumoto Y., Tada F., Matsuura B., Abe M., Hiasa Y. (2015). Nonalcoholic fatty liver disease: Portal hypertension due to outflow block in patients without cirrhosis. Radiology.

[59] Ryou M., Stylopoulos N., Baffy G. (2020). Nonalcoholic fatty liver disease and portal hypertension. Explor. Med..

[60] Paternostro R., Kwanten W.J., Reiberger T. (2023). Portal hypertension is a key determinant of the risk for liver-related events in non-alcoholic fatty liver disease. J. Hepatol..

[61] Schuppan D., Afdhal N.H. (2008). Liver cirrhosis. Lancet.

[62] Bernardi M., Moreau R., Angeli P., Schnabl B., Arroyo V. (2015). Mechanisms of decompensation and organ failure in cirrhosis: From peripheral arterial vasodilation to systemic inflammation hypothesis. J. Hepatol..

[63] Henin G., Loumaye A., Leclercq I.A., Lanthier N. (2023). Myosteatosis: Diagnosis, pathophysiology and consequences in metabolic dysfunction-associated steatotic liver disease. JHEP Rep..

[64] Maurice J., Manousou P. (2018). Non-alcoholic fatty liver disease. Clin. Med..

[65] Masuoka H.C., Chalasani N. (2013). Nonalcoholic fatty liver disease: An emerging threat to obese and diabetic individuals. Ann. N. Y. Acad. Sci..

[66] Targher G., Day C.P., Bonora E. (2010). Risk of cardiovascular disease in patients with nonalcoholic fatty liver disease. N. Engl. J. Med..

[67] Pennisi G., Di Marco V., Buscemi C., Mazzola G., Randazzo C., Spatola F., Craxì A., Buscemi S., Petta S. (2021). Interplay between non-alcoholic fatty liver disease and cardiovascular risk in an asymptomatic general population. J. Gastroenterol. Hepatol..

[68] Mantovani A., Csermely A., Petracca G., Beatrice G., Corey K.E., Simon T.G., Byrne C.D., Targher G. (2021). Non-alcoholic fatty liver disease and risk of fatal and non-fatal cardiovascular events: An updated systematic review and meta-analysis. Lancet Gastroenterol. Hepatol..

[69] Pais R., Giral P., Khan J.F., Rosenbaum D., Housset C., Poynard T. (2016). Fatty liver is an independent predictor of early carotid atherosclerosis. J. Hepatol..

[70] Lonardo A., Nascimbeni F., Mantovani A., Targher G. (2018). Hypertension, diabetes, atherosclerosis and NASH: Cause or consequence?. J. Hepatol..

[71] Driessen S., Francque S.M., Anker S.D., Cabezas M.C., Grobbee D.E., Tushuizen M.E., Holleboom A.G. (2025). Metabolic dysfunction–associated steatotic liver disease and the heart. Hepatology.

[72] Williams B., Mancia G., Spiering W., Agabiti Rosei E., Azizi M., Burnier M., Clement D.L., Coca A., De Simone G., Dominiczak A. (2018). 2018 ESC/ESH Guidelines for the management of arterial hypertension. Eur. Heart J..

[73] Mancia G., Kreutz R., Brunström M., Burnier M., Grassi G., Januszewicz A., Muiesan M.L., Tsioufis K., Agabiti-Rosei E., Algharably E.A.E. (2023). 2023 ESH Guidelines for the management of arterial hypertension The Task Force for the management of arterial hypertension of the European Society of Hypertension: Endorsed by the International Society of Hypertension (ISH) and the European Renal Association (ERA). J. Hypertens..

[74] Ciardullo S., Grassi G., Mancia G., Perseghin G. (2022). Nonalcoholic fatty liver disease and risk of incident hypertension: A systematic review and meta-analysis. Eur. J. Gastroenterol. Hepatol..

[75] Hallsworth K., Hollingsworth K.G., Thoma C., Jakovljevic D.G., MacGowan G.A., Anstee Q.M., Taylor R. (2013). Cardiac structure and function are altered in adults with NAFLD. J. Hepatol..

[76] Bonapace S., Perseghin G., Molon G., Canali G., Bertolini L., Zoppini G., Targher G. (2012). NAFLD is associated with left ventricular diastolic dysfunction in type 2 diabetes. Diabetes Care.

[77] Mantovani A., Pernigo M., Bergamini C., Bonapace S., Lipari P., Pichiri I., Targher G. (2015). NAFLD is independently associated with early LV diastolic dysfunction. PLoS ONE.

[78] Chang W., Wang Y., Sun L., Yu D., Li Y., Li G. (2019). Evaluation of left atrial function in type 2 diabetes mellitus patients with nonalcoholic fatty liver disease by two-dimensional speckle tracking echocardiography. Echocardiography.

[79] Dong Y., Cui H., Sun L., Wang Y., Li Y., Chang W., Li G., Huang D. (2020). Assessment of left ventricular function in type 2 diabetes mellitus patients with non-alcoholic fatty liver disease using three-dimensional speckle-tracking echocardiography. Anatol. J. Cardiol..

[80] Cameli M., Mondillo S., Galderisi M., Mandoli G.E., Ballo P., Nistri S., Capo V., D’Ascenzi F., D’Andrea A., Esposito R. (2017). Speckle tracking echocardiography: Roadmap for meas-urement and clinical use. G. Ital. Cardiol..

[81] Goland S., Shimoni S., Zornitzki T., Knobler H., Azoulai O., Lutaty G., Melzer E., Orr A., Caspi A., Malnick S. (2006). Cardiac abnormalities as a new manifestation of nonalcoholic fatty liver disease. J. Clin. Gastroenterol..

[82] Fotbolcu H., Yakar T., Duman D., Karaahmet T., Tigen K., Basaran Y. (2010). Impairment of left ventricular systolic and diastolic function in patients with non-alcoholic fatty liver disease. Cardiol. J..

[83] Fallo F., Dalla Pozza A., Sonino N., Lupia M., Tona F., Federspil G., Ermani M., Catena C., Soardo G., Di Piazza L. (2009). Non-alcoholic fatty liver disease is associated with left ventricular diastolic dysfunction in essential hypertension. Nutr. Metab. Cardiovasc. Dis..

[84] Karabay C.Y., Kocabay G., Kalaycı A., Zehir R., Güler A., Oduncu V. (2014). Impaired left ventricular mechanics in nonalcoholic fatty liver disease. Eur. J. Gastroenterol. Hepatol..

[85] VanWagner L.B., Wilcox J.E., Colangelo L.A., Lloyd-Jones D.M., Carr J.J., Lima J.A., Lewis C.E., Rinella M.E., Shah S.J. (2015). Association of nonalcoholic fatty liver disease with subclinical myocardial remodeling and dysfunction. Hepatology.

[86] VanWagner L.B., Wilcox J.E., Ning H., Lewis C.E., Carr J.J., Rinella M.E., Shah S.J., Lima J.A., Lloyd-Jones D.M. (2020). Longitudinal association of NAFLD with changes in myocardial structure and function. J. Am. Heart Assoc..

[87] Granér M., Nyman K., Siren R., Pentikäinen M.O., Lundbom J., Hakkarainen A., Lauerma K., Lundbom N., Nieminen M.S., Taskinen M.R. (2014). Ectopic fat depots and left ventricular function in nondiabetic men with nonalcoholic fatty liver disease. Circ. Cardiovasc. Imaging.

[88] Gruber M., Almasri M., Abdulredha R., Tecar I., Leucuta D.-C., Popa S.-L., Dumitrascu D.L., Ismaiel A. (2025). When the Liver Echoes to the Heart: Assessing Subclinical Cardiac Dysfunction in NAFLD Using Speckle Tracking Echocardiography—A Systematic Review and Meta-Analysis. Biomedicines.

[89] Alp H., Karaarslan S., Selver Eklioğlu B., Atabek M.E., Altın H., Baysal T. (2013). Association between nonalcoholic fatty liver disease and cardiovascular risk in obese children and adolescents. Can. J. Cardiol..

[90] Pacifico L., Di Martino M., De Merulis A., Bezzi M., Osborn J.F., Catalano C., Chiesa C. (2014). Left ventricular dysfunction in obese children and adolescents with nonalcoholic fatty liver disease. Hepatology.

[91] Singh G.K., Vitola B.E., Holland M.R., Sekarski T., Patterson B.W., Magkos F., Klein S. (2013). Alterations in ventricular structure and function in obese adolescents with nonalcoholic fatty liver disease. J. Pediatr..

[92] Mantovani A., Morandin R., Sani E., Fiorio V., Shtembari E., Bonapace S., Petta S., Polyzos S.A., Byrne C.D., Targher G. (2025). MASLD Is Associated With an Increased Long-Term Risk of Atrial Fibrillation: An Updated Systematic Review and Meta-Analysis. Liver Int..

[93] Mantovani A., Dauriz M., Sandri D., Bonapace S., Zoppini G., Tilg H., Byrne C.D., Targher G. (2019). Association between non-alcoholic fatty liver disease and risk of atrial fibrillation in adult individuals: An updated meta-analysis. Liver Int..

[94] Haghbin H., Gangwani M.K., Ravi S.J.K., Perisetti A., Aziz M., Goyal H. (2020). Nonalcoholic fatty liver disease and atrial fibrillation: Possible pathophysiological links and therapeutic interventions. Ann. Gastroenterol..

[95] Zhang S., Liang H., Liu J., Huang Z., Shi X., Zhu Y. (2025). Association between inflammatory, metabolic, and hepatic fibrosis–related biomarkers and atrial fibrillation in older patients with metabolic dysfunction–associated steatotic liver disease. BMC Geriatr..

[96] Wang M., Zhou B.G., Zhang Y., Ren X.F., Li L., Li B., Ai Y.W. (2022). Association Between Non-alcoholic Fatty Liver Disease and Risk of Stroke: A Systematic Review and Meta-Analysis. Front. Cardiovasc. Med..

[97] Parikh N.S., VanWagner L.B., Elkind M.S.V., Gutierrez J. (2019). Association between nonalcoholic fatty liver disease with advanced fibrosis and stroke. J. Neurol. Sci..

[98] Perseghin G., Lattuada G., De Cobelli F., Esposito A., Belloni E., Ntali G., Ragogna F., Canu T., Scifo P., Del Maschio A. (2008). Increased mediastinal fat and impaired left ventricular energy metabolism in young men with newly found fatty liver. Hepatology.

[99] Chung G.E., Lee J.-H., Lee H., Kim M.K., Yim J.Y., Choi S.-Y., Kim Y.J., Yoon J.-H., Kim D. (2018). Nonalcoholic fatty liver disease and advanced fibrosis are associated with left ventricular diastolic dysfunction. Atherosclerosis.

[100] European Association for the Study of the Liver (EASL), European Association for the Study of Diabetes (EASD), European Association for the Study of Obesity (EASO) (2024). EASL-EASD-EASO Clinical Practice Guidelines on the Management of Metabolic Dysfunction-Associated Steatotic Liver Disease (MASLD). Obes. Facts.

[101] Monserrat-Mesquida M., Bouzas C., García S., Mateos D., Casares M., Ugarriza L., Gómez C., Sureda A., Tur J.A. (2025). Two-Year Mediterranean Diet Intervention Improves Hepatic Health in MASLD Patients. Foods.

[102] Kim A., Krishnan A., Hamilton J.P., Woreta T.A. (2021). The Impact of Dietary Patterns and Nutrition in Nonalcoholic Fatty Liver Disease. Gastroenterol. Clin. N. Am..

[103] Arita V.A., Cabezas M.C., Hernández Vargas J.A., Trujillo-Cáceres S.J., Mendez Pernicone N., Bridge L.A., Raeisi-Dehkordi H., Dietvorst C.A., Dekker R., Uriza-Pinzón J.P. (2025). Effects of Mediterranean diet, exercise, and their combination on body composition and liver outcomes in metabolic dysfunction-associated steatotic liver disease: A systematic review and meta-analysis of randomized controlled trials. BMC Med..

[104] Von Loeffelholz C., Roth J., Coldewey S.M., Birkenfeld A.L. (2021). The role of physical activity in nonalcoholic and metabolic dysfunction associated fatty liver disease. Biomedicines.

[105] Zong G., Mao W., Wen M., Cheng X., Liu G. (2025). Association of sleep patterns and disorders with metabolic dysfunction-associated steatotic liver disease and liver fibrosis in contemporary American adults. Ann. Hepatol..

[106] Macavei B., Baban A., Dumitrascu D.L. (2016). Psychological factors associated with NAFLD/NASH: A systematic review. Eur. Rev. Med. Pharmacol. Sci..

[107] Chang K.C., Kuo F.C., Yang C.Y., Yang C.T., Ou H.T., Kuo S. (2024). Non-alcoholic fatty liver disease risk with GLP-1 receptor agonists and SGLT-2 inhibitors in type 2 diabetes: A nationwide nested case-control study. Cardiovasc. Diabetol..

[108] Wu J.Y., Hsu W.H., Kuo C.C., Tsai Y.W., Liu T.H., Huang P.Y., Chuang M.H., Hung K.C., Yu T., Lai C.C. (2025). A retrospective analy-sis of combination therapy with GLP-1 receptor agonists and SGLT2 inhibitors versus SGLT2 inhibitor monothera-py in patients with MASLD. Nat. Commun..

[109] Vincent R.K., Williams D.M., Evans M. (2020). A look to the future in non-alcoholic fatty liver disease: Are glucagon-like pep-tide-1 analogues or sodium-glucose co-transporter-2 inhibitors the answer?. Diabetes Obes. Metab..

[110] Liu Q.K. (2024). Mechanisms of action and therapeutic applications of GLP-1 and dual GIP/GLP-1 receptor agonists. Front. Endocrinol..

[111] Mantovani A., Petracca G., Csermely A., Beatrice G., Targher G. (2020). Sodium-Glucose Cotransporter-2 Inhibitors for Treatment of Nonalcoholic Fatty Liver Disease: A Meta-Analysis of Randomized Controlled Trials. Metabolites.

[112] Kuo C.C., Chuang M.H., Li C.H., Tsai Y.W., Huang P.Y., Kuo H.T., Lai C.C. (2025). Glucagon-Like Peptide-1 Receptor Agonists and Liver Outcomes in Patients With MASLD and Type 2 Diabetes. Aliment. Pharmacol. Ther..

[113] Li M., Hu J., Han Chin Y., Chew H.S.J., Wang W. (2025). Comparative effectiveness of GLP-1 receptor agonists and dual agonists in the treatment of patients with metabolic dysfunction associated steatohepatitis: A systematic review and me-ta-analysis. Front. Endocrinol..

[114] Fiorucci S., Urbani G. (2025). Tirzepatide for metabolic dysfunction-associated steatohepatitis: Results from phase II clinical trials and perspectives. Expert. Opin. Investig. Drugs.

[115] Li Y., Sun W., Liu H., Ruan X.Z. (2025). Tirzepatide, a dual GIP/GLP-1 receptor agonist, alleviates metabolic dysfunction-associated steatotic liver disease by reducing the expression of CD36 and OBP2A. Genes. Dis..

[116] Bu T., Sun Z., Pan Y., Deng X., Yuan G. (2024). Glucagon-Like Peptide-1: New Regulator in Lipid Metabolism. Diabetes Metab. J..

[117] Shimizu M., Suzuki K., Kato K., Jojima T., Iijima T., Murohisa T., Iijima M., Takekawa H., Usui I., Hiraishi H. (2019). Evaluation of the effects of dapagliflozin, a sodium-glucose co-transporter-2 inhibitor, on hepatic steatosis and fi-brosis using transient elastography in patients with type 2 diabetes and non-alcoholic fatty liver disease. Diabetes Obes. Metab..

[118] Chen X., Zhang X., Xiang X., Fang X., Feng S. (2024). Effects of glucagon-like peptide-1 receptor agonists on cardiovascular outcomes in high-risk type 2 diabetes: A systematic review and meta-analysis of randomized controlled trials. Diabetol. Metab. Syndr..

[119] Marcondes-de-Castro I.A., Marinho T.S., Aguila M.B., Mandarim-de-Lacerda C.A. (2026). Tirzepatide enhances liver structural integrity by promoting mitochondrial dynamics and mitophagy via PINK1/PRKN and SIRT3/NRF2 pathways in an obese-diabetic-menopausal mouse model. Tissue Cell.

[120] Tamilwanan S., Aziz Z., Rong L.Y., Bitar A.N., Zarzour R.H.A., Alshehade S.A. (2025). Efficacy of GLP-1 receptor agonists and dual GLP-1/GIP receptor agonists in managing MALFD: A meta-analysis of randomized controlled trials. BMC Gas-Troenterol..

[121] Singh A., Sohal A., Batta A. (2024). GLP-1, GIP/GLP-1, and GCGR/GLP-1 receptor agonists: Novel therapeutic agents for metabolic dysfunction-associated steatohepatitis. World J. Gastroenterol..

[122] Tornea D.A., Goldis C., Isaic A., Motofelea A.C., Sima A.C., Ciocarlie T., Crintea A., Diaconescu R.G., Motofelea N., Goldis A. (2026). The Effect of GLP-1 Agonists on Patients with Metabolic-Associated Steatotic Liver Disease: A Systematic Review and Meta-Analysis. Pharmaceutics.

[123] Loomba R., Hartman M.L., Lawitz E.J., Vuppalanchi R., Boursier J., Bugianesi E., Yoneda M., Behling C., Cummings O.W., Tang Y. (2024). Tirzepatide for metabolic dysfunction-associated steatohepatitis with liver fibrosis. N. Engl. J. Med..

[124] Mirarchi L., Amodeo S., Citarrella R., Licata A., Soresi M., Giannitrapani L. (2022). SGLT2 Inhibitors as the Most Promising Influencers on the Outcome of Non-Alcoholic Fatty Liver Disease. Int. J. Mol. Sci..

[125] Zhang W., Hu H. (2025). The emerging therapeutic promise of SGLT2 inhibitors in metabolic dysfunction-associated steatotic liver disease. Dig. Dis. Sci..

[126] Suki M., Imam A., Amer J., Milgrom Y., Massarwa M., Hazou W., Tiram Y., Perzon O., Sharif Y., Sackran J. (2025). SGLT2 Inhibitors in MASLD (Metabolic Dysfunc-tion-Associated Steatotic Liver Disease) Associated with Sustained Hepatic Benefits, Besides the Cardiometabolic. Pharmaceuticals.

[127] Procyk G., Jaworski J., Gąsecka A., Filipiak K.J., Borovac J.A. (2024). Metabolic dysfunction-associated steatotic liver disease - A new indication for sodium-glucose Co-transporter-2 inhibitors. Adv. Med. Sci..

[128] Targher G., Byrne C.D., Tilg H. (2024). MASLD: A systemic metabolic disorder with cardiovascular and malignant complications. Gut.

[129] Ahmed A.E., Alhufayyan N.S., Qadri H.F., Atiah A.F., Alhazmi W.M., Alhazemi N.T., Zalah H.A., Nasser F.M., Monajid R.M., Aljuhani M.G. (2025). Efficacy of Sodium-Glucose Cotransporter 2 Inhibitors in Patients With Type 2 Diabetes Mellitus and Nonalcoholic Fatty Liver Disease: A Systematic Review of Randomized Controlled Trials. Cureus.

[130] Shi M., Zhang H., Wang W., Zhang X., Liu J., Wang Q., Wang Y., Zhang C., Guo X., Qiao Q. (2023). Effect of dapagliflozin on liver and pancreatic fat in patients with type 2 diabetes and non-alcoholic fatty liver disease: Randomized controlled trial. J. Diabetes Its Complicat..

[131] Weng M.-T., Yang P.-J., Liu P.-F., Chang C.-H., Lee H.-S., Sheu J.-C., Nien H.-C. (2025). Effects of dapagliflozin on liver steatosis in patients with nonalcoholic fatty liver disease: A randomized controlled trial. Hepatol. Int..

[132] Sun L., Deng C., Gu Y., He Y., Yang L., Shi J. (2022). Effects of dapagliflozin in patients with nonalcoholic fatty liver disease: A systematic review and meta-analysis of randomized controlled trials. J. Clin. Transl. Hepatol..

[133] Fukada H., Kon K., Yaginuma R., Uchiyama A., Morinaga M., Ishizuka K., Fukuhara K., Okubo H., Suzuki S., Nojiri S. (2025). Effectiveness and risks of dapagliflozin in treatment for metabolic dysfunction-associated steatotic liver disease with type 2 diabetes: A randomized controlled trial. Front. Med..

[134] Alsarkhi L.N., Eda S., Mohammed Abdul R.H., Hussaini H., Fadeyi O., Chaudhari S.S., Rauf M.Q., Allahwala D. (2025). Empagliflozin Treatment for Non-alcoholic Fatty Liver Disease in Type 2 Diabetes Patients: A Systematic Review and Meta-Analysis of Randomized Controlled Trials. Cureus.

[135] Chehrehgosha H., Sohrabi M.R., Ismail-Beigi F., Malek M., Reza Babaei M., Zamani F., Ajdarkosh H., Khoonsari M., Fallah A.E., Khamseh M.E. (2021). Empagliflozin Improves Liver Steatosis and Fibrosis in Patients with Non-Alcoholic Fatty Liver Disease and Type 2 Diabetes: A Randomized, Double-Blind, Placebo-Controlled Clinical Trial. Diabetes Ther..

[136] Hiruma S., Shigiyama F., Kumashiro N. (2023). Empagliflozin versus sitagliptin for ameliorating intrahepatic lipid content and tissue-specific insulin sensitivity in patients with early-stage type 2 diabetes with non-alcoholic fatty liver disease: A prospective randomized study. Diabetes Obes. Metab..

[137] Cheung K.S., Ng H.Y., Hui R.W.H., Lam L.K., Mak L.Y., Ho Y.C., Tan J.T., Chan E.W., Seto W.K., Yuen M.F. (2024). Effects of empagliflozin on liver fat in patients with metabolic dysfunction-associated steatotic liver disease without diabetes mellitus: A randomized, double-blind, placebo-controlled trial. Hepatology.

[138] Tang X., Zhang H., Wang X., Yang D. (2022). Empagliflozin for the treatment of non-alcoholic fatty liver disease: A meta-analysis of randomized controlled trials. Afr. Health Sci..

[139] Khaliq A., Badshah H., Shah Y., Rehman I.U., Khan K.U., Ming L.C., Cheng M.H. (2024). The effect of ertugliflozin in patients with nonalcoholic fatty liver disease associated with type 2 diabetes mellitus: A randomized controlled trial. Medicine.

[140] Gallo S., Calle R.A., Terra S.G., Pong A., Tarasenko L., Raji A. (2020). Effects of Ertugliflozin on Liver Enzymes in Patients with Type 2 Diabetes: A Post-Hoc Pooled Analysis of Phase 3 Trials. Diabetes Ther..

[141] Khaliq A., Badshah H., Shah Y. (2025). Combination therapy with vitamin E and ertugliflozin in patients with non-alcoholic fatty liver disease and type 2 diabetes mellitus: A randomized clinical trial. Ir. J. Med. Sci..

[142] Nishimiya N., Tajima K., Imajo K., Kameda A., Yoshida E., Togashi Y., Aoki K., Inoue T., Nakajima A., Utsunomiya D. (2021). Effects of Canagliflozin on Hepatic Steatosis, Visceral Fat and Skeletal Muscle among Patients with Type 2 Diabetes and Non-alcoholic Fatty Liver Disease. Inter. Med..

[143] Itani T., Ishihara T. (2018). Efficacy of canagliflozin against nonalcoholic fatty liver disease: A prospective cohort study. Obes. Sci. Pract..

[144] Lee K.W., Devaraj N.K., Ching S.M., Veettil S.K., Hoo F.K., Deuraseh I., Soo M.J. (2021). Effect of SGLT-2 Inhibitors on Non-alcoholic Fatty Liver Disease among Patients with Type 2 Diabetes Mellitus: Systematic Review with Meta-analysis and Trial Sequential Analysis of Randomized Clinical Trials. Oman Med. J..

[145] Lu M., Zhao Y., Liu Z., Zhang Y., Liu J. (2025). Canagliflozin Alleviates Metabolic Dysfunction-Associated Steatotic Liver Disease via Mitochondrial Protection and Enhanced Mitophagy. Hepatol. Res..

[146] Siafarikas C., Kapelios C.J., Papatheodoridi M., Vlachogiannakos J., Tentolouris N., Papatheodoridis G. (2024). Sodium-glucose linked transporter 2 inhibitors in liver cirrhosis: Beyond their antidiabetic use. Liver Int..

[147] Abu-Hammour M.N., Abdel-Razeq R., Vignarajah A., Khedraki R., Sims O.T., Vigneswaramoorthy N., Chiang D.J. (2025). Sodium-Glucose Cotransporter 2 Inhibitors and Serious Liver Events in Patients with Cirrhosis. JAMA Netw. Open.

[148] Kuchay M.S., Krishan S., Mishra S.K., Farooqui K.J., Singh M.K., Wasir J.S., Bansal B. (2018). Effect of empagliflozin on liver fat in patients with type 2 diabetes and nonalcoholic fatty liver disease: A randomized controlled trial (E-LIFT Trial). Diabetes Care.

[149] Seko Y., Sumida Y., Tanaka S., Mori K., Taketani H., Ishiba H., Hara T., Okajima A., Umemura A., Yoneda M. (2017). Effect of sodium glucose cotransporter 2 inhibitors on fatty liver in patients with type 2 diabetes mellitus and non-alcoholic fatty liver disease. Hepatol. Res..

[150] McGuire D.K., Shih W.J., Cosentino F., Charbonnel B., Cherney D.Z.I., Dagogo-Jack S., Pratley R., Greenberg M., Wang S., Huyck S. (2021). Association of SGLT2 Inhibitors with Cardiovascular and Kidney Outcomes in Patients with Type 2 Diabetes: A Meta-analysis. JAMA Cardiol..

[151] Cherney D.Z.I., Dekkers C.C.J., Barbour S.J., Cattran D., Abdul Gafor A.H., Greasley P.J., Laverman G.D., Lim S.K., Di Tanna G.L., Reich H.N. (2020). Effects of the SGLT2 inhibitor dapagliflozin on proteinuria in non-diabetic kidney disease: A prespecified analysis from DAPA-CKD. Lancet Diabetes Endocrinol..

[152] McMurray J.J.V., Solomon S.D., Inzucchi S.E., Køber L., Kosiborod M.N., Martinez F.A., Ponikowski P., Sabatine M.S., Anand I.S., Bělohlávek J. (2019). Dapagliflozin in patients with heart failure and reduced ejection fraction. N. Engl. J. Med..

[153] Packer M., Anker S.D., Butler J., Filippatos G., Pocock S.J., Carson P., Januzzi J., Verma S., Tsutsui H., Brueckmann M. (2020). Cardiovascular and renal outcomes with empagliflozin in heart failure. N. Engl. J. Med..

[154] Taheri H., Malek M., Ismail-Beigi F., Zamani F., Sohrabi M., Reza Babaei M., Khamseh M.E. (2020). Effect of Empagliflozin on Liver Steatosis and Fibrosis in Patients with Non-Alcoholic Fatty Liver Disease Without Diabetes: A Randomized, Dou-ble-Blind, Placebo-Controlled Trial. Adv. Ther..

[155] Wang Y., Zhou Y., Prud’homme G.J., Wang Q. (2025). Efficacy of GLP-1-based therapies on metabolic dysfunction-associated steatotic liver disease and metabolic dysfunction-associated steatohepatitis: A systematic review and meta-analysis. J. Clin. Endocrinol. Metab..

[156] Morris S.M., Armstrong M.J., Newsome P.N. (2022). Safety and Efficacy of Glucagon-like Peptide 1 Receptor Agonists in Pa-tients with Cirrhosis. Clin. Gastroenterol. Hepatol..

[157] Lv X., Dong Y., Hu L., Lu F., Zhou C., Qin S. (2020). Glucagon-like peptide-1 receptor agonists (GLP-1 RAs) for the management of nonalcoholic fatty liver disease (NAFLD): A systematic review. Endocrinol. Diabetes Metab..

[158] Abushamat L.A., Shah P.A., Eckel R.H., Harrison S.A., Barb D. (2024). The Emerging Role of Glucagon-Like Peptide-1 Receptor Agonists for the Treatment of Metabolic Dysfunction-Associated Steatohepatitis. Clin. Gastroenterol. Hepatol..

[159] Abdelmalek M.F., Harrison S.A., Sanyal A.J. (2024). The role of glucagon-like peptide-1 receptor agonists in metabolic dys-function-associated steatohepatitis. Diabetes Obes. Metab..

[160] Armstrong M.J., Gaunt P., Aithal G.P., Barton D., Hull D., Parker R., Hazlehurst J.M., Guo K., Abouda G., Aldersley M.A. (2016). Liraglutide safety and efficacy in patients with non-alcoholic steatohepatitis (LEAN): A multicentre, double-blind, randomised, placebo-controlled phase 2 study. Lancet.

[161] Mantovani A., Petracca G., Beatrice G., Csermely A., Lonardo A., Targher G. (2021). Glucagon-Like Peptide-1 Receptor Agonists for Treatment of Nonalcoholic Fatty Liver Disease and Nonalcoholic Steatohepatitis: An Updated Me-ta-Analysis of Randomized Controlled Trials. Metabolites.

[162] Kuchay M.S., Krishan S., Mishra S.K., Choudhary N.S., Singh M.K., Wasir J.S., Kaur P., Gill H.K., Bano T., Farooqui K.J. (2020). Effect of dulaglutide on liver fat in patients with type 2 diabetes and NAFLD: Randomised controlled trial (D-LIFT trial). Diabetologia.

[163] Liu C., Xin Y., Huang Y., Xu L., Zhou R., Wang Y., Wang W. (2025). Reduction of Hepatic Fat Content by Dulaglutide for the Treatment of Diabetes Mellitus: A Two-Centre Open, Single-Arm Trial. Endocrinol. Diabetes Metab..

[164] Shantaram D., Rima X.Y., Bradley D., Liu J.Z., Wright V.P., Amari A., Jalilvand A., Rottinghaus J., Fernandes J.M., Smith A.J. (2025). The GLP-1 receptor agonist dulaglutide attenuates hepatic steatosis in obesity via a weight-independent mechanism. Diabetes.

[165] Fang L., Zeng H., Li J., Liu J. (2024). Effects of GLP-1 receptor agonists on the degree of liver fibrosis and CRP in non-alcoholic fatty liver disease and non-alcoholic steatohepatitis: A systematic review and meta-analysis. Prim. Care Diabetes.

[166] Kan R., Wang S., Meng X., Guo Y., Li D., Yu X. (2025). The impact of semaglutide on liver outcomes in patients with or at risk of MASH: A dose and duration response meta-analysis of randomized trials. Diabetol. Metab. Syndr..

[167] Zhu K., Kakkar R., Chahal D., Yoshida E.M., Hussaini T. (2023). Efficacy and safety of semaglutide in non-alcoholic fatty liver disease. World J. Gastroenterol..

[168] Kuo C.-C., Chuang M.-H., Li C.-H., Huang P.-Y., Kuo H.-T., Lai C.-C. (2025). Semaglutide and the risk of adverse liver outcomes in patients with nonalcoholic fatty liver disease and type 2 diabetes: A multi-institutional cohort study. Hepatol. Int..

[169] Dusilová T., Kovář J., Laňková I., Thieme L., Hubáčková M., Šedivý P., Pajuelo D., Burian M., Dezortová M., Miklánková D. (2024). Semaglutide treatment effects on liver fat content in obese subjects with metabolic-associated steatotic liver disease (MASLD). J. Clin. Med..

[170] Bansal M.B., Patton H., Morgan T.R., Carr R.M., Dranoff J.A., Allen A.M. (2025). Semaglutide therapy for metabolic dysfunction-associated steatohepatitis: November 2025 updates to AASLD Practice Guidance. Hepatology.

[171] Yabut J.M., Drucker D.J. (2023). Glucagon-like Peptide-1 Receptor-based Therapeutics for Metabolic Liver Disease. Endocr. Rev..

[172] Meyhöfer S.M., Cariou B., Cercato C., Colhoun H.M., Deanfield J., Long M.T., Jeppesen O.K., Lincoff A.M., Lingvay I., Plutzky J. (2026). Semag-lutide on liver fibrosis and heart outcomes in patients at high risk of liver fibrosis: A prespecified analysis of the SE-LECT randomized trial. Nat. Med..

[173] Elsaid M.I., Li N., Firkins S.A., Rustgi V.K., Paskett E.D., Acharya C., Reddy K.R., Chiang C.W., Mumtaz K. (2024). Impacts of glucagon-like peptide-1 receptor agonists on the risk of adverse liver outcomes in patients with metabolic dysfunction-associated steatotic liver disease cirrhosis and type 2 diabetes. Aliment. Pharmacol. Ther..

[174] Mantovani A., Morandin R., Fiorio V., Lando M.G., Stefan N., Tilg H., Byrne C.D., Targher G. (2025). Glucagon-Like Peptide-1 Receptor Agonists Improve MASH and Liver Fibrosis: A Meta-Analysis of Randomised Controlled Trials. Liver Int..

[175] Hartman M.L., Sanyal A.J., Loomba R., Wilson J.M., Nikooienejad A., Bray R., Karanikas C.A., Duffin K.L., Robins D.A., Haupt A. (2020). Effects of novel dual GIP and GLP-1 receptor agonist tirzepatide on biomarkers of nonalcoholic steatohepatitis in patients with type 2 diabetes. Diabetes Care.

[176] Sanyal A.J., Newsome P.N., Kliers I., Østergaard L.H., Long M.T., Kjær M.S., Cali A.M.G., Bugianesi E., Rinella M.E., Roden M. (2025). ESSENCE Study Group. Phase 3 Trial of Semaglutide in Metabolic Dysfunction-Associated Steatohepatitis. N. Engl. J. Med..

[177] Marso S.P., Daniels G.H., Brown-Frandsen K., Kristensen P., Mann J.F., Nauck M.A., Nissen S.E., Pocock S., Poulter N.R., Ravn L.S. (2016). Liraglutide and Cardiovascular Outcomes in Type 2 Diabetes. N. Engl. J. Med..

[178] Butler P.C., Elashoff M., Elashoff R., Gale E.A. (2013). A critical analysis of the clinical use of incretin-based therapies: Are the GLP-1 therapies safe?. Diabetes Care.

[179] Miyamoto Y., Honda A., Yokose S., Nagata M., Miyamoto J. (2023). The Effects of SGLT2 Inhibitors on Liver Cirrhosis Patients with Refractory Ascites: A Literature Review. J. Clin. Med..

[180] Allen S.L., Quinlan J.I., Dhaliwal A., Armstrong M.J., Elsharkawy A.M., Greig C.A., Lord J.M., Lavery G.G., Breen L. (2021). Sarcopenia in chronic liver disease: Mechanisms and countermeasures. Am. J. Physiol. Gastrointest. Liver Physiol..

[181] Bhandarkar A., Bhat S., Kapoor N. (2025). Effect of GLP-1 receptor agonists on body composition. Curr. Opin. Endocrinol. Diabetes Obes..

[182] Zinman B., Wanner C., Lachin J.M., Fitchett D., Bluhmki E., Hantel S., Mattheus M., Devins T., Johansen O.E., Woerle H.J. (2015). Empagliflozin, Cardiovascular Outcomes, and Mortality in Type 2 Diabetes. N. Engl. J. Med..

[183] Wiviott S.D., Raz I., Bonaca M.P., Mosenzon O., Kato E.T., Cahn A., Silverman M.G., Zelniker T.A., Kuder J.F., Murphy S.A. (2019). Dapagliflozin and Cardiovascular Outcomes in Type 2 Diabetes. N. Engl. J. Med..

[184] Abbas M.S., Dandamudi M., Rehman T., Faizan M.A., Zahra I., Mojica J.C., Ahmed M.R., Bai K., Shakeel I., Shahzad Z. (2025). Efficacy of SGLT2 inhibitors in non-diabetic non-alcoholic fatty liver disease: A systematic review and meta-analysis. J. Diabetes Metab. Disord..

